# Ancient Ancestry Informative Markers for Identifying Fine-Scale Ancient Population Structure in Eurasians

**DOI:** 10.3390/genes9120625

**Published:** 2018-12-12

**Authors:** Umberto Esposito, Ranajit Das, Syakir Syed, Mehdi Pirooznia, Eran Elhaik

**Affiliations:** 1 Department of Animal and Plant Sciences, University of Sheffield, Sheffield S10 2TN, UK; u.esposito@sheffield.ac.uk (U.E.); syakir.syed@gmail.com (S.S.); 2 Manipal University, Manipal Centre for Natural Sciences (MCNS), Manipal, Karnataka 576104, India; ranajit.das@manipal.edu; 3 Bioinformatics and Computational Biology, National Heart Lung and Blood Institute, National Institutes of Health, Bethesda, MD 20892, USA; mehdi.pirooznia@nih.gov

**Keywords:** ancient DNA, ancient ancestry informative markers, population structure, principal component analysis, admixture mapping, primordialism

## Abstract

The rapid accumulation of ancient human genomes from various areas and time periods potentially enables the expansion of studies of biodiversity, biogeography, forensics, population history, and epidemiology into past populations. However, most ancient DNA (aDNA) data were generated through microarrays designed for modern-day populations, which are known to misrepresent the population structure. Past studies addressed these problems by using ancestry informative markers (AIMs). It is, however, unclear whether AIMs derived from contemporary human genomes can capture ancient population structures, and whether AIM-finding methods are applicable to aDNA. Further the high missingness rates in ancient—and oftentimes haploid—DNA can also distort the population structure. Here, we define ancient AIMs (aAIMs) and develop a framework to evaluate established and novel AIM-finding methods in identifying the most informative markers. We show that aAIMs identified by a novel principal component analysis (PCA)-based method outperform all of the competing methods in classifying ancient individuals into populations and identifying admixed individuals. In some cases, predictions made using the aAIMs were more accurate than those made with a complete marker set. We discuss the features of the ancient Eurasian population structure and strategies to identify aAIMs. This work informs the design of single nucleotide polymorphism (SNP) microarrays and the interpretation of aDNA results, which enables a population-wide testing of primordialist theories.

## 1. Introduction

### 1.1. Towards High-Resolution Population Models Using Ancient Samples

Over the past decade, genomic techniques have been reshaping our fundamental understanding of human prehistory and origins [1]. Ancient DNA (aDNA) human genomes have assisted in investigations of population structure, human migration, human adaptation, agricultural lifestyle, and disease co-evolution [2]. Ancient genome studies have already accelerated progress in the search for genetic variations underlying the inheritance of adaptations and forensics traits. Recently, Cassidy et al. [3] tested the allelic association of cystic fibrosis and hemochromatosis in ancient Irish samples, expanding genetic epidemiology onto ancient genomes. Such studies can potentially identify new risk factors for rare diseases.

### 1.2. Next Generation Sequencing Technologies to Study Ancient DNA

Whole genome sequencing and single nucleotide polymorphism (SNP) microarrays are the two leading approaches to aDNA sequencing. Although the former is preferable as it provides more data, by late 2017, only a quarter of the 1100 sequenced ancient genomes were whole genomes. The vast majority of these genomes (762) were captured by SNP microarrays [2], mainly the Human Origins [4] and Illumina 610-Quad arrays [5,6]—neither of which were designed for ancient humans—making it particularly challenging to identify and control for ancient population structure. Future microarrays, dedicated to aDNA, will thereby need a reliable set of polymorphic markers that can be used to differentiate ancient populations whose population structure was shown to vary over time [7].

Single nucleotide polymorphism genotyping microarrays were originally developed to detect phenotype–genotype associations in association mapping, admixture mapping, identity by descent mapping, and similar studies. It was not until later that SNP microarrays were employed in population genetic studies aimed at inferring population structure through principal component analyses (PCAs), ADMIXTURE-like programs, and other tools aimed at predicting group membership. It soon became clear that the allele frequency spectrum obtained through microarrays is more skewed for some populations than for other ones due to the choice of SNP panels [8]. The Human Origins and various Illumina microarrays (including the Illumina Human 660W-Quad, which is very similar to the Illumina 610-Quad array) were shown to distort the population structure for modern day populations compared to larger genomic databases and underreport the biodiversity compared to microarrays customized for population genetics [9,10], which results in an ascertainment bias.

### 1.3. The Problems of Ascertainment Bias and Population Stratification in Ancient DNA

Any inference of identity in archeological studies is fraught with difficulties. Carbon dating requires extracting organic material from fossil bones and authenticating it as composed of degraded proteins; this process is highly susceptible to contamination, which yields erroneous estimates [11]. The identification of ‘cultures’ from archaeological remains and their association with past population groups is also inadequate [12]. Population genetic studies suffer from similar problems due to ascertainment bias, which can distort measures of human diversity, bias population genetic inferences, and alter the conclusions in unexpected ways [13]. Ascertainment bias is a major concern in genetic, biomedical, and evolutionary studies, particularly in the absence of an established population structure model for either modern-day or ancient populations.

The difficulties related to establishing an acceptable population model are partially due to our incomplete knowledge of human population biodiversity in the past and present. Often, modern-day populations are assumed to be the parental populations of the modern-day population of interest, which results in population stratification. This problem arises due to differences in the allele frequencies of unknown case/control subpopulations due to separate demographic histories (not biological processes). A misunderstanding of the population structure necessitates mismatched cases and controls, which introduces genetic heterogeneity into the analysis that can lead to spurious associations and obscure the true association [14]. Thereby, the phenotypes of interest (e.g., risk loci or drug response) may differ between these subpopulations and bias the association analyses by generating false positives [15]. These problems have been well-known for a long time, and statistical remedies have been proposed [16]; however, they were all tailored for modern-day data, and do not address the conceptual problems. It is now clear that population models should consider aDNA data and the unique challenges they pose, such as, haploidy that reduces the biodiversity of the samples and high missingness, which precludes comparing individuals on the same marker set [1].

### 1.4. The Use of Ancestry Informative Markers in Genetics

Past studies have resolved, to a large extent, the problems faced in DNA analyses with ancestry informative markers (AIMs). Ancestry informative markers are SNPs that exhibit large variation in minor allele frequencies (MAF) among populations. Over the past two decades, geneticists have scoured genomes for these patterns, and to date produced numerous AIM sets to determine an individual’s ancestry, detect stratification in biomedical studies, infer geographic structure, find risk loci in a candidate region, and localize biogeographical origins (e.g., [9,10,17,18,19]). Ancestry informative marker panels can delineate population structure in a cost-effective manner by detecting variation in individual ancestry that can confound methods such as Mendelian randomization trials, association analyses, and forensic investigations by increasing false positive results or reducing power [20].

Although initially preferred due to the high cost of sequencing (which has decreased with time) AIMs are still frequently used in forensics, carrier screening, and biogeography in both microarrays (e.g., [9,21]) and whole genomic data [22]. Admixture mapping is another powerful method to map phenotypic variation or diseases that show differential risk by ancestry. The mapping takes advantage of higher densities of genetic variants and extensions to admixed populations, which exhibit strong differences in prevalence across populations [23]. Therefore, it is necessary to have a large number of AIMs throughout the genome to allow for the inference of local chromosomal ancestry blocks.

Despite their high prevalence, it has never been clear which AIMs should be used. All AIM panels have limitations [24] and it is unknown whether the established AIMs would be informative for aDNA studies. The characteristics of ideal AIMs remain contentious, with some authors preferring common SNPs (minor allele frequency >1%) [25], SNPs with high fixation index (*F_ST_*) [26], SNPs with high pairwise MAF between populations [24], or SNPs that satisfy several criteria. Consequently, AIMs do not overlap across studies. Of the 21 AIM datasets reviewed by Pakstis et al. [27], the union of SNPs consisted of 1397 AIMs appearedof which only 46 occurred in three to six panels. Finally, studies typically show that AIMs can separate populations or broadly classify individuals into subcontinental populations rather than showing that AIMs can capture the population structure of the complete SNP set or allow fine-population mapping. Given the uncertainties surrounding AIMs, their potential incompatibility for capturing ancient structure and admixtures, and the challenges imposed by aDNA data, it is unclear whether, if at all, AIM-finding methods or AIMs can be utilized to study ancient population structures.

### 1.5. Ancient Ancestry Informative Markers to Define Ancient Population Structure

**Figure 1:**
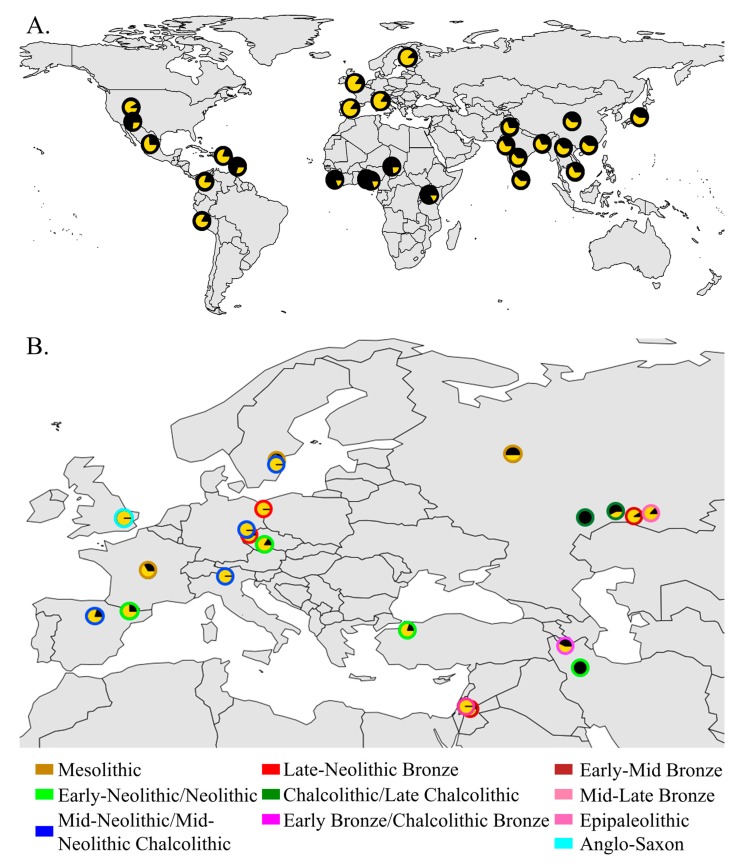
Geographic distribution of the highly differentiated rs7896530 in modern-day (**A**) and ancient (**B**) populations. The geographic distributions of the T (black) and G (yellow) alleles were obtained from the Geography of Genetic Variants Browser [33] and Table S1, respectively.

**Figure 2:**
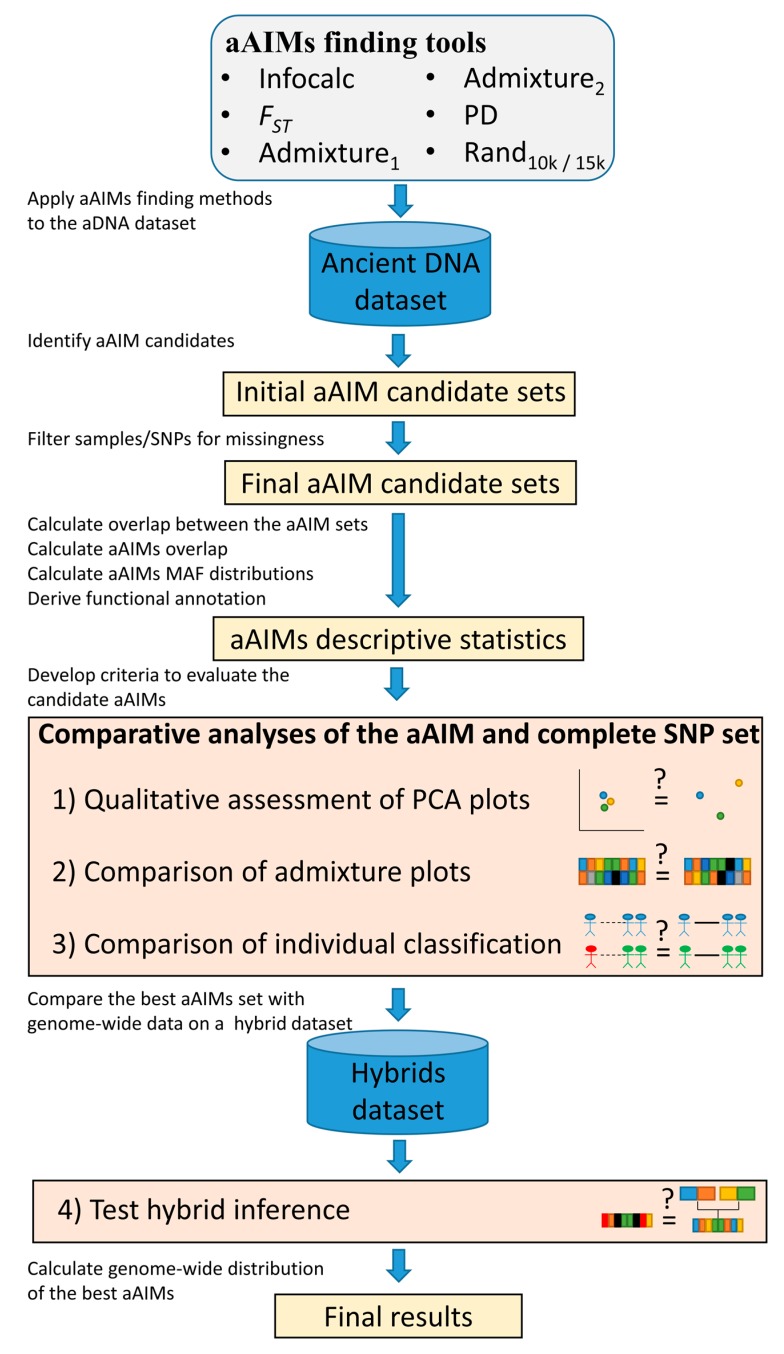
A workflow to identify and evaluate the accuracy of ancient ancestry informative markers (aAIM)-finding algorithms compared to each other as well as to the complete single nucleotide polymorphism (SNP) (CSS) set. We adopted four criteria to evaluate how well the aAIM candidates captured the population structure depicted by the CSS. First, we qualitatively compared the dispersal of genomes obtained from a principal component analysis (PCA) to that of the CSS. Second, we compared the Euclidean distances between the admixture proportions of each genome and those obtained from the CSS. To avoid inconsistencies between the SNP sets, we used admixture components obtained through a *supervised* ADMIXTURE (see methods). Third, we tested which aAIMs classified individuals to populations most accurately. Finally, we evaluated the ability of the top performing method to identify admixed individuals against the CSS. aDNA: ancient DNA.

aDNA, aDNA allows for the construction of AIM panels from the parental populations of modern-day people and can refine population structure estimates. To overcome some of the aforementioned problems with aDNA data, we defined ancient ancestry informative markers (aAIMs) as SNPs that vary in their MAF across ancient populations (Figure 1) and attempted to identify and validate the first autosomal aAIMs in order to improve the inference of ancient population structure. In the absence of aAIM-finding tools, we selected several established AIM-finding tools: two existing AIM-finding algorithms (Infocalc [28] and *F*_ST_-based algorithm [29] that employed Wright’s *F*_ST_ [30]) and developed three novel admixture and PCA-based algorithms. Both methods have characteristics that have been reported to be beneficial in measuring genetic distances in population genetic studies (e.g., [16,31,32]), and were expected to be useful in identifying AIMs. Since AIM-finding tools were never tested on aDNA, it is necessary to first compare their ability in finding informative markers, which can differentiate ancient populations. For that, we interrogated a comprehensive dataset of 302 ancient genomes from Europe, the Middle East, and North Eurasia, spanning time periods from 14,000 through to 1500 years ago, and that were sequenced using both microarray and whole genome technology. These genomes were grouped into 21 populations based on geographical and temporal information (Table S1). Each population was then further divided into subpopulations based on the genetic similarity between the genomes in terms of their admixture profile. To test how well the aAIM candidates, as identified by various tools, capture the population structure and identify admixed individuals, we first derived summary statistics using these aAIM candidates. Then, we compared the performances of the best aAIM set with the complete SNP set in classifying individuals to populations and identifying two-way admixed individuals (Figure 2). Our current study offers a methodological framework to evaluate AIMs, contrasts different AIM-finding strategies, reports the first set of aAIMs, and demonstrates that in some cases, they provide more reliable predictions than the complete SNP set.

## 2. Materials and Methods

### 2.1. Ancient Data Collection

Genomic data were obtained from 11 publications depicting 302 ancient genomes (Table S1). In the case of sequence data, sequence reads were aligned to the human reference assembly (UCSC hg19-http://genome.ucsc.edu/) using the Burrows Wheeler Aligner (BWA version 0.7.15) [34], allowing two mismatches in the 30-base seed. Alignments were then imported to binary (bam) format, sorted, and indexed using SAMtools (version 1.3.1) [35]. Picard (version 2.1.1) (http://picard.sourceforge.net/) and MarkDuplicates were used to remove reads with identical outer mapping coordinates (which are likely PCA artifacts). The Genome Analysis Toolkit RealignerTargetCreator module (GATK version 3.6) [36,37] was used to generate SNP and small insertion/deletion (InDel) calls for the data within the targeted regions only. GATK InDelRealigner/BaseRecalibrator was then used for local read realignment around known InDels and for the base quality score recalibration of predicted variant sites based on dbSNP 138 and 1000 Genomes known sites, resulting in corrections for base reported quality. The recalibration was followed by SNP/InDel calling with the GATK HaplotypeCaller. Variants were filtered for a minimum confidence score of 30 and a minimum mapping quality of 40. At the genotype level, all of the genotypes that had a genotype depth of less than four (GD < 4) or a genotype quality score less than 10 (GQ < 10) were removed from the dataset by setting them as missing in the VCF. GATK DepthofCoverage was used to re-examine coverage following the realignment. VCFtools (version 0.1.14) [38] were used to convert the VCF file to PLINK format [39]. The final dataset comprised of 150,278 autosomal SNPs from 302 aDNA genomes (Table S1; Additional file 1 in Supplementary Materials). Eight aDNA genomes (I0247, I1584, ATP9, IR1, Kostenki14, MA1, and Ust Ishim) without any country/region designation were omitted in the closest population determination calculations. The genomes were classified into 21 populations, based on their sampling country/region and era.

### 2.2. Data Analyses

#### 2.2.1. The Genetic Structure Canvas of Ancient Eurasian Genomes

The population structure of the ancient genomes was described using PCA implemented in PLINK v1.9 [39]. Ancient genomes and SNPs with over 90% missingness were removed. We also applied the model-based clustering methods implemented in ADMIXTURE v1.3 [40]. Minor allele frequency was calculated using PLINK (--maf command). The MAF for modern-day populations was calculated from the 1000 Genomes populations (ALL.2of4intersection.20100804.genotypes) [41]. The percentage of rare and novel variants and other functional information were obtained through the Variant Effect Predictor (VEP).

#### 2.2.2. Identifying aAIMs Using Multiple Methods

We applied two established and three novel methods to detect aAIM candidates as follows:

1. **Infocalc** v1.1 [28], determines the amount of information that multiallelic markers provide of an individual’s ancestry by calculating the informativeness (*I*) of each marker separately and ranking the SNPs by their informativeness. Infocalc determines *I* based on the mathematical expression described in Rosenberg et al. (2003). We compared the performances (Figure 2) of the top 5000, 10,000, 15,000, and 20,000 most informative markers (results not shown). The 15,000 dataset outperformed all of the other datasets, and was selected for further analyses.
2. ***F_ST_***. Wright’s fixation indices (*F*_ST_) [30] measures the degree of differentiation among populations, which was potentially arising due to the genetic structure within populations. Given a set of populations (Table S1), we employed PLINK v1.9 [39] to estimate *F_ST_* separately for all the markers using the --fst command alongside --within flag. Due to the high fragmentation of the data, *F_ST_* values could only be calculated for 46% of the dataset. We compared the performances (Figure 2) of 5000, 10,000, 15,000, and 20,000 SNPs with the highest *F_ST_* values (results not shown). The 15,000 dataset outperformed all of the other datasets, and was selected for further analyses.
3. **Admixture_1_**. This method assumes that aAIMs have high allelic frequencies in certain subpopulations that drive the differentiation of admixture components. Analyzing ADMIXTURE’s output file (P file) for *K* = 10, we identified the markers (rows) that had high allele frequency (>0.9) in only one admixture component (columns). Comparing the number of high-MAF SNPs in all of the columns, we selected 9309 from the five columns with the highest number of such SNPs.
4. **Admixture_2_**. This method assumes that aAIMs embody both high allelic frequencies in certain subpopulations, and that the high variance between these allelic frequencies differentiates the admixture components. Analyzing ADMIXTURE’s output file for *K* of 10, we identified 11,418 SNPs showing high variance (≥0.04) and a high allele frequency range (maxima–minima ≥ 0.65) between the admixture components.
5. **Principal Component-derived (PD)**. This method assumes that AIMs can replicate the population structure of subpopulations represented by the variation in the first two PCs. This is an interactive PC-based approach that identifies the smallest set of markers necessary to capture the population structure of a group of individuals, as captured by the complete SNP set (CSS). More specifically, for each population group (Table S1) in which at least 100 SNPs were available, we carried out PCA after all of the SNPs with high missingness (>0.05) were excised. If the population group had insufficient SNPs, we relaxed the missingness threshold by an additional 0.05, although 0.05 were sufficient for almost all of the groups. We then scored the SNPs by their informativeness, as in [42], and used the top 100 most informative SNPs to plot the individuals on a scatter plot using PC1 and PC2 as axes. We visually compared the plot to that obtained from the CSS (Figure S11). If the plots were dissimilar, we repeated the analysis using an additional 100 top-scored SNPs until either the plots exhibited high similarity or a threshold of 2000 SNPs was reached. In this manner, we identified the minimum number of the most informative SNPs that were needed to replicate the PCA results of the CSS. We were unable to complete the analyses for three populations due to the small number of individuals. The PD method is available on https://github.com/eelhaik/PCA-derived-aAIMs. On average, 861 SNPs were collated per population group. Overall, the dataset comprised 13,027 SNPs.

To compare the prediction accuracy of the aAIMs subsets, two control datasets (Rand_10k_ and Rand_15k_) were generated by randomly sampling 10,000 and 15,000 SNPs from the CSS, respectively. The aAIMs identified by all of these methods are available as Additional File 2 in Supplementary Materials.

#### 2.2.3. Classifying Individuals into Populations from Genomic Data

Following the reported success of the admixture-based method, which employs AIMs to describe and classify individuals to populations [17,43,44,45], we sought to develop an analogous method that employs aAIMs.

Identifying ancient admixture components: To avoid over-fitting, and since some of the methods employ ADMIXTURE, we sought to identify admixture components in a small cohort of diverse individuals. For that, we selected 100 random ancient genomes and removed six because of insufficient data (>95% missingness). To those, we added 20 Han Chinese and 20 Yoruba modern genomes from the 1000 Genomes Project [41]. We then applied an *unsupervised* ADMIXTURE with the parameter *K* ranging from 8 to 13. Although we were unable to find a single *K* when culturally related genomes exhibited homogeneous admixture patterns, the most robust population substructure was found for the *K* value of 10. Two more admixture components were obtained by separately analyzing the Spanish and German genomes, which appeared indistinguishable in the original analysis, along with five Yoruba genomes. We observed very little admixture of the ancient individuals with the Han and Yoruba. Overall, we identified 10 admixture components in ancient genomes, corresponding to the allele frequencies of 10 hypothetical populations. Similar to Elhaik et al. [17], we simulated 15 samples to represent each hypothetical population by generating 30 alleles whose MAF values corresponded to the MAF of each population, and assigning those genotypes to the simulated individuals. The putative ancestral ancient populations are available in Additional File 3 in the Supplementary Materials.

Relabeling populations: Initially, the labels from the corresponding literature were used to classify individuals to population. The consistency of these labels with data was evaluated by carrying out a *supervised* ADMIXTURE analysis on the genomic data combined with the 150 putative ancient ancestral individuals. Due to the high similarity of the admixture patterns between individuals of different groups living in similar periods or entire groups (e.g., Neolithic individuals from Hungary and those from Germany), we relabeled some of the population to reduce the number of populations and create more genomically homogeneous populations. For instance, Natufian and Neolithic samples from Jordan are grouped into the label Levant Epipaleolithic Neolithic. Overall, we identified 21 populations whose labels are of the form “area_time period”. In the case of the Caucasus label, all of the samples from Iran (except Iran_HotuIIIb) were excavated in the Zagros Mountains, south of the Caucasus. Given their admixture similarity with Armenians and Georgians from the same periods and their proximity to the Caucasus, this area was labeled Caucasus. Iran_HotuIIIb was found in a more eastern region, just below the southeastern edge of the Caspian Sea, and given its similarity to Georgians and other Iranians, it was included in the group Caucasus Mesolithic Neolithic.

Genomically defining reference populations: For each population with *N_P_* > 4, where *N_P_* is the number of individuals assigned to that population, individuals were grouped into subclusters through an iterative process that uses the *k*-means clustering technique paired with multiple pairwise *F*-tests. Iterations ran over the number of *k* subclusters [*k* = 2, ..., *N_P_*/2]. At each iteration *i*, *k*-means was used to identify the *k* subclusters; then, the *F*-test was applied on each pair of subclusters to test whether they were significantly different (*p* < 0.05). If two clusters were significantly different from all of the pairs at iteration *i*, the process repeats for *i* + 1 until at least one pair violates the condition, in which case the optimal number of *k* subclusters or reference populations within that population is the number of subclusters that did not violate the condition.

Assigning individuals to populations: We developed an admixture-based classifier that was not sensitive to the exclusion of random groups of individuals or the inclusion of large numbers of individuals from admixed groups, and was trained on a third of the data. Using a *supervised* ADMIXTURE, we calculated the admixture proportions of the individuals in relation to the putative ancient ancestral populations. Population assignment was then made based on the minimal Euclidean distance between the admixture components of each genome and those of the reference populations. The assignment accuracy was measured against the population classification (Table S1).

#### 2.2.4. Assessing Admixture Mapping

Creating hybrid individuals: We selected 15 individuals from five populations that showed homogeneity in their admixture components (Figure S4) and randomly sampled 120 pairs. Since selecting random alleles from each parent was problematic due to the high missingness of the data, we randomly selected half the genotypes of each parent to form 120 “offspring” or hybrid genomes. Each hybrid had three SNP sets: the CSS, PD aAIMs, and a random SNP set of the size of PD aAIMs with SNPs selected randomly for each hybrid.

Assessing admixture accuracy: Following [43,45,46], we applied a *supervised* ADMIXTURE to the three SNP sets of each hybrid.

## 3. Results

### 3.1. Depicting Ancient Population Structure

**Figure 3:**
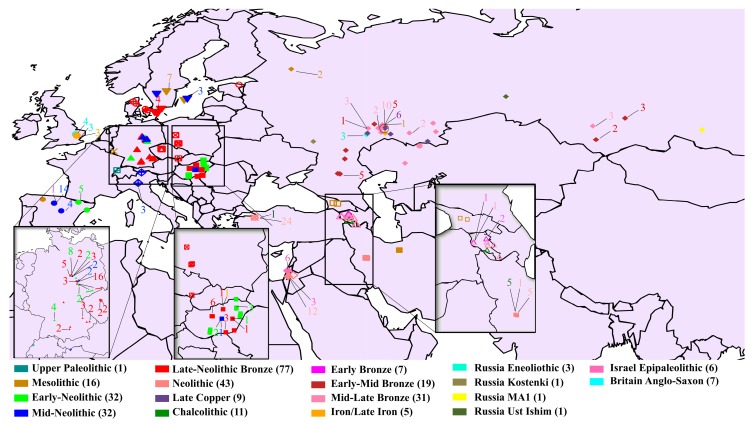
Geographical locations of the ancient genomes. The shapes designate the country of origin of the genomes and their colors designate the era. The total number of ancient genomes from each era is noted. Insets show densely sampled regions.

We constructed a dataset of 150,278 autosomal SNPs from 302 ancient genomes classified into 21 populations from Europe, the Middle East, and North Eurasia, and dated to time periods spanning from 14,000 years ago through to 1500 years ago (Figure 3, Table S1). These samples were chosen in order to obtain a broad temporal and geographical coverage. Nonetheless, due to the limited availability of ancient genomes, our dataset was not uniform over time and space. For instance, there were 57 Central European genomes from the Late Neolithic to the Bronze Age, but populations such as Mesolithic Central and Western Europeans, Bronze Age Jordanians, Chalcolithic Russians, and Mesolithic Russians comprised only of three genomes each. The population labels that we used corresponded directly with those from the published papers; in some cases, they were left unchanged, while in others cases we merged groups with similar admixture profiles in order to create broader, but homogenous populations.

Missingness varied greatly within the samples, as well as within the markers. The sample-based missingness ranged from 0.05% (KK1) to 99.2% (I1951), with a mean of 54%. Similarly, missingness also varied among the populations, with Levantine Epipaleolithic and Neolithic genomes having the highest missingness (*n* = 19, *µ* = 90, *σ* = 16) and Mesolithic Swedish genomes having the lowest (*n* = 8, *µ* = 29, *σ* = 27). The SNP-based missingness ranged from 30% to 98%, with an mean of 54%.

Principal component analysis (PCA) of the ancient genomes substantiated previous observations of a Europe–Middle East contrast along the vertical principal component (PC1) and parallel clines (PC2) in both Europe and the Middle East (Figure S1). Genomes from the Epipaleolithic and Neolithic Levantine clustered at one extreme of the Near East–Europe cline with some overlapping with Neolithic Turkish and Central European genomes. Neolithic Iranians were clustered between Central European genomes. While ancient Spanish, Armenian, Central European Union (EU), and British genomes occupied the intermediate position of Near Eastern and North Eurasian genomes, Russian and Swedish genomes clustered at the end of the Near East–Europe cline.

Our *unsupervised* ADMIXTURE analysis with a range of splits (*K*) (Figure S2) found that no choice of *K* minimized the cross-validation error (CVE) (Figure S3), as expected in the analysis of monder-day populations, probably because the high noise and missingness in the data prevented the CVE from stabilizing. At *K* = 10 (Figure S4), multiple genomes (e.g., Britain Iron Saxon, Mesolithic Neolithic Caucasus population, Bronze Age Jordanian, Epipaleolithic Levantine, Chalcolithic, Mesolithic and Early Mid Bronze Russian, Early Neolithic Spanish, Mesolithic and Mid Neolithic Swedish, and Neolithic Turkish) appeared to be homogeneous in relation to their population and exhibited a distinct allelic frequency profile of admixture components. For these reasons, we decided to choose *K* = 10 as the optimal value. Furthermore, in this case, putative ancient ancestral components, such as the *Early Neolithic European* (brown) and the *Russia Mid Late Bronze* (magenta), which were predominantly found among European genomes, could be identified. Except for their predominance in Neolithic Turkish genomes, these two components also exist in most Neolithic Central Europeans. Some 20–30% of Central European genomes have discernible fractions of *Europe Late Neolithic–Early Bronze* (navy-blue) and *Russia Mid–Late Bronze* (deep-pink) components, respectively. Two components (cyan and dark purple) appeared sporadically in a few populations, which was likely due to noise.

### 3.2. Identifying and Describing the Ancient Ancestry Informative Markers Candidates

We developed a framework to identify and evaluate the efficacy of aAIM candidates in capturing ancient population structure and allowing admixture mapping (Figure 2). Ancient ancestry informative marker candidates were identified using five methods (Figure 2). Similar to the CSS, genomes and SNPs with over 90% missingness were removed, leaving each dataset with 223–263 genomes (Table S2). Furthermore, 310 SNPs without data were removed from the Rand_10k_ dataset. The final number of aAIM candidates is shown in Table S3. Overlapping aAIMs between the methods are remarkably small and range from 560 (Rand_10k_ and Admixture_1_) to 2160 (Admixture_1_ and Admixture_2_). Interestingly, Infocalc and *F_ST_*, which are often used together, share only ~10% of their aAIM candidates. The PD method shares 13.7% of its aAIMs with *F_ST_* and ~10% with Infocalc.

Comparing the properties of the aAIM candidates (Figure S5a), we found that Infocalc prioritized SNPs with the lowest MAF (45% of the aAIMs have MAF < 0.1) and *F_ST_* captured the aAIMs with a high frequency of low–mid MAFs. By contrast, PD and the admixture-based methods exhibited higher frequencies of high MAF SNPs, with Admixture_2_ having the highest proportion of high MAF aAIMs (75% of the aAIMS have MAF >0.4). Remarkably, the MAF distributions exhibited a similarity with modern populations (Figure S5b), though, with fewer alleles in the lowest MAF bins for all the methods. Unsurprisingly, most of the aAIM variants were non-functional (94.6–96.3%) and varied little from the CSS’s annotation (Table S4).

### 3.3. Comparative Testing of Ancient Ancestry Informative Marker Candidates

The accuracy of the aAIMs was evaluated using four criteria and by comparing each method against both CSS and two random SNP sets of sizes that approximated the number of aAIM candidates. We first calculated the PCA for each SNP set and compared the population dispersion along the primary two axes. Similarly to the CSS (Figure S1), all the methods depicted the Europe–Middle East contrast (PC1) and parallel clines (PC2) in the European genomes so that ancient Jordanian, Levantine, Turkic, and Spanish genomes clustered at one extreme of the Near East–Europe cline, whereas the genomes from Russia and Sweden clustered at the other end (Figure S6). However, similar as with the random sets, Infocalc and *F_ST_* did not separate Levantine and Turkic individuals from each other. Infocalc also merged the Caucasus individuals with central Europeans. The admixture-based methods and PD clearly separated all of the ancient populations, similar to the CSS or more discernably, in the case of Scandinavians and Russians.

**Figure 4:**
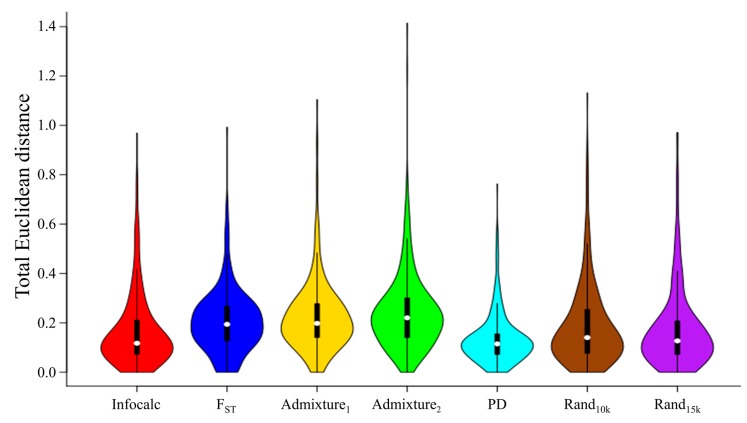
A comparison of the Euclidean distances (Δ) between the admixture proportions of the ancient genomes obtained from the CSS and those obtained from the aAIM sets using violin plots. Lower distances indicate high genetic similarity between the admixture proportions obtained using two different SNP sets.

Secondly, we quantitatively assessed which dataset produced the closest admixture signature to that of the CSS (Figure S4). For that, we calculated the admixture proportions in relation to the 10 putatively ancient ancestral populations that we obtained with the CSS (Figure S7), and then computed their Euclidean distances Figure S8 to their counterparts obtained with the CSS (Figure 4). The PD aAIMs led to significantly shorter Euclidean distances (*μ* = 0.13, *σ* = 0.1, *n* = 302) compared to those obtained from the other aAIMs (Welch *t*-test: Infocalc (*t* = 2.99, *p*-value = 0.002), *F_ST_* (*t* = 7.32, *p*-value = 8.5 × 10^−13^), Admixture_1_ (*t* = 8.71, *p*-value = 2.2 × 10^−16^), Admixture_2_ (*t* = 9.89, *p*-value = 2 × 10^−16^), Rand_10k_ (*t* = 4.59, *p*-value = 5 × 10^−6^), and Rand_15k_ (*t* = 3.27, *p*-value = 0.001)). Infocalc’s aAIMs produced the second-shortest distances from the CSS (*μ* = 0.17, *σ* = 0.15); however, these differences in the distances compared to those obtained with the two random datasets were not statistically significant (Welch *t*-test: Rand_10k_ (*t* = 1.56, *p*-value = 0.12) and Rand_15k_ (*t* = 0.33, *p*-value = 0.77), respectively), suggesting that Infocalc was unable to capture the population structure. *F_ST_*-derived AIMs (*μ* = 0.2, *σ* = 0.13) performed significantly worse than the Rand_15k_ aAIMs (Welch *t*-test, *t* = 2.89, *p*-value 0.004), and similar to the Rand_10k_ aAIMs (Welch *t*-test, *t* = 1.5, *p*-value = 0.13). Finally, the two admixture-based datasets performed the worst out of all the methods (*μ*_1_ = 0.22, *σ*_1_ = 0.15 and *μ*_2_ = 0.24, *σ*_1_ = 0.16) and significantly worse than the two random datasets (Welch *t*-test: Admixture_1_ [Rand_10k_ *t* = 2.99, *p*-value = 0.002] and [Rand_15k_ *t* = 4.35, *p*-value = 1.6 × 10^−5^]; Admixture_2_ [Rand_10k_ *t* = 4.34, *p*-value = 1.7 × 10^−5^] and [Rand_15k_ *t* = 5.65, *p*-value = 2.5 × 10^−8^]).

Thirdly, we assessed which aAIMs dataset allowed classification of individuals into population groups most accurately. An admixture-based population classifier was applied to the admixture proportions produced by all of the datasets, and their accuracy was compared to that of the CSS (76 ± 5%) and the known population classification (Table S1). The mean classification accuracy per population ranged from 3% (*F_ST_*) to 61% (PD), with the PD outperforming all of the other methods (Table 1). In other words, ~13k (8%) of the SNPs are sufficiently informative to classify individuals to populations with 80% of the accuracy of the CSS. In nine out of 21 population groups (22% of the individuals), PD-based classification was similar or more accurate than the CSS. All other methods performed similarly or worse than the two random SNP sets (Rand_10k_ = 42 ± 5% and Rand_15k_ = 50 ± 5%), with Infocalc (50 ± 6%) outperforming the remaining methods. Of note is the poor performance of *F_ST_* aAIMs, which indicates its unsuitability for aDNA data. As expected, high missingness was associated with incorrect predictions (Figure S9). For example, the low-coverage, low-quality Britain Anglo-Saxon genomes proved challenging for all of the methods (0–40%), but predicted correctly with the CSS (100%).

### 3.4. Inference of Admixed Samples

The last criterion used to evaluate the accuracy of the aAIMs was to test whether they can identify hybrid individuals. Due to the high accuracy of the PD aAIMs in classifying individuals into populations, when compared to the alternative datasets, we decided to focus on aAIMs identified by the PD. Figure S10 illustrates the genome-wide distribution of PD aAIMs. To assess whether these aAIMs can identify hybrid individuals, ancient individuals were hybridized to form 120 mixed individuals who were represented in three datasets: CSS, PD aAIMs, and a random SNP set of the size of PD aAIMs (Table 2).

The genetic admixture distances between the hybrid individuals that were generated using the CSS and PD aAIMs were significantly smaller (*µ* = 0.05, *σ* = 0.04) than the genetic admixture distances between the CSS and the random SNP set (*µ* = 0.45, *σ* = 0.15, Welch *t*-test *p*-values = 2.2 × 10^−8^) and those between the PD and the random SNP set (*µ* = 0.43, *σ* = 0.15, Welch *t*-test *p*-values = 1.9 × 10^−8^). Thus, we demonstrated that PD aAIMs can be used for studying admixed individuals and can be potentially used in future admixture mapping involving aDNA.

## 4. Discussion

Questions of identity and primordialism are at the center of scientific and public debate. Until recently, charting the emergence of agriculture, the spread of languages, and the rise and decline of cultures were topics dominated by archeologists. The emergence of aDNA allows paleogeneticists to delve into this debate with a discordant set of assumptions about biology and identity [47]. This was not unforeseen, as population genetic analyses excel at identifying individual differences, which can inform archeologically contended subjects such as migration and the degree of admixture or population replacements. However, aDNA analyses also require destroying genetic material, sometimes irrevocably, which makes them impossible to replicate. It is therefore crucial to develop a robust genetic methodology that uses population genetic principles to examine the assumptions made by both archeologists and paleogeneticists. It is reasonable to expect that many of the tools employed to study modern-day genomes will need to be adapted to the four-dimensional environment facilitated by aDNA.

Ancestry informative markers are some of the most useful tools in addressing population, biomedical, forensics, and evolutionary questions that remain in use today [9,48,49,50]. However, it is unclear to what extent known AIMs are applicable to ancient genomic data, which are characterized by high missingness and haploidy [1].

In this study, we defined aAIMs (Figure 1) and sought to identify them using various methods. The number of aAIM candidates detected by each method ranged from 9,000 to 15,000. These numbers are of the same magnitude as large AIMs studies (e.g., [51,52]) and reasonable, provided that there is potential relatedness of the ancient Eurasian populations and the near absence of heterozygote markers in the data. To find which of the aAIM candidate sets produced by each method best represent the true population structure, we used the CSS as a benchmark for qualitative and quantitative comparisons.

Identifying the ideal AIM set that would be both small and include redundancies (in the case of sequencing failure), capture the population structure, and allow the identification of admixed individuals is one of the challenges of population genetics. We showed that the aAIMs identified through the PD method outperformed all other methods, in agreement with previous studies that tested PCA-based methods [25]. In forty percent of the populations, classifications made by the PD method were more accurate than those made using the CSS (Table 1), which highlights the limitations of using markers indiscriminately. This is not surprising, since not all the markers are equally informative, and less informative markers (e.g., exonic markers) may mask the population structure, resulting in the misclassification of populations. The notion of “more is better” is, hence particularly misguided with aDNA that harbors a multi-layered population structure in a poor set of markers. The application of the PD aAIMs for admixture mapping, combined with tools that can homogenize cases and controls [16], enables the carrying out of future association studies on aDNA samples (e.g., [3]). Further investigations with additional data may identify formerly common markers associated with those disease that with time became rare and undetectable.

The use of PCA to infer population structure is controversial [53,54,55,56], and its use as a clustering method has been criticized [16]. We note that the PD method employs PCA only to produce and replicate a population structure profile of certain subpopulations based on various sets of markers and does not make claims that the PCA-derived profiles represent the true genetic distances between individuals.

Surprisingly, Infocalc and *F_ST_* that are commonly used to identify AIMs [18] and are reported to perform well [57], have oftentimes underperformed random SNP selections. Not only was *F_ST_* already shown to be particularly small within continental populations [58], but these methods may be particularly sensitive to aDNA data that are both haploid and have high missingness (Figure S9). We also found no relationships between the performances of MAF and aAIMs (Figure S5). Enrichment for high or low MAF SNPs did not guarantee success, although the PD harbored more common SNPs than most of the underperforming methods.

Our study has several limitations. We studied an uneven number of Eurasian populations from various times and locations, causing a skew toward markers that predict central European populations from the Late Neolithic and Bronze Age. A modest attempt to reduce this bias was made by including modern-day African and Asian populations; however, more comprehensive analyses should be made when more global genomes are available from consecutives eras. Second, the aAIMs were calculated independently by each method with individual populations considered independent, although the PCA and ADMIXTURE plots indicate that central European populations may not be independent. Finally, due to the high missingness of the data, it is likely that our study missed informative markers that could improve the classification accuracy in newly sequenced populations. Therefore, our framework and methods must be applied again when more comprehensive aDNA datasets are available.

## 5. Conclusions

The use of ancient genomes in research is in its infancy, and is expected to intensify and expand to new fields as more data become available. One of the main advantages of aDNA is that it widens the number of ancestry types and makes them multi-faceted, requiring fine-tuned molecular utilities to depict ancestry over time. AIMs are some of the most effective tools that have spear-headed population genetics over the past two decades and are ancillary to the challenge of understanding population structure. Here, we defined aAIMs, proposed a framework to evaluate AIM-finding methods, demonstrated the accuracy of a novel aAIM-finding method, and reported the most successful set of aAIMs. Future analyses may benefit from using our framework, methods, and aAIMs in order to refine ancient population structure models and examine primordialist theories.

## Figures and Tables

**Table 1:** Accuracy in classifying individuals to populations using the aAIM candidates. The total number of individuals (*n*) per population are reported in column two. Columns three to eight show the number of individuals correctly predicted to their populations and, in brackets, the corresponding population percentage. Columns seven and eight effectively represent a random number of 10000 and 15000 SNPs, respectively. Mean and standard error for each SNP set are provided in the last row.

Population	*n*	CSS	PD	*F_ST_*	Infocalc	Admixture_1_	Admixture_2_	Rand_10k_	Rand_15k_
Britain Iron Saxon	10	10 (100)	4 (40)	0 (0)	0 (0)	0 (0)	0 (0)	1 (10)	3 (30)
Caucasus Chalcolithic Bronze	22	21 (95)	8 (36)	0 (0)	12 (55)	6 (27)	4 (18)	13 (59)	9 (41)
Caucasus Mesolithic Neolithic	9	6 (67)	7 (78)	0 (0)	6 (67)	1 (11)	7 (78)	4 (44)	4 (44)
Central EU Early Neolithic	26	17 (65)	14 (54)	4 (15)	18 (69)	4 (15)	5 (19)	14 (54)	18 (69)
Central EU Late Neolithic Bronze	57	16 (28)	17 (30)	19 (33)	19 (33)	13 (23)	21 (37)	25 (44)	21 (37)
Central EU Mid Neolithic Chalc	6	2 (33)	3 (50)	0 (0)	3 (50)	3 (50)	3 (50)	2 (33)	2 (33)
Central North EU Late Neol Bronz	20	18 (90)	9 (45)	0 (0)	6 (30)	0 (0)	5 (25)	4 (20)	6 (30)
Central Western EU Mesolithic	3	3 (100)	2 (67)	0 (0)	3 (100)	0 (0)	0 (0)	1 (33)	3 (100)
Italy Mid Neolithic Chalcolithic	4	4 (100)	3 (75)	0 (0)	1 (25)	1 (25)	0 (0)	1 (25)	1 (25)
Jordan Bronze	3	3 (100)	2 (67)	0 (0)	0 (0)	2 (67)	3 (100)	1 (33)	2 (67)
Levant Epipaleolithic Neolithic	19	7 (37)	6 (32)	0 (0)	9 (47)	8 (42)	7 (37)	4 (21)	7 (37)
Russia Chalcolithic	3	2 (67)	3 (100)	0 (0)	1 (33)	0 (0)	2 (67)	1 (33)	1 (33)
Russia Early Mid Bronze	19	19 (100)	15 (79)	0 (0)	10 (53)	0 (0)	18 (95)	10 (53)	14 (74)
Russia Late Chalcolithic	9	6 (67)	6 (67)	0 (0)	5 (56)	0 (0)	1 (11)	3 (33)	3 (33)
Russia Mesolithic	3	2 (67)	2 (67)	0 (0)	2 (67)	0 (0)	1 (33)	2 (67)	2 (67)
Russia Mid Late Bronze	22	15 (68)	16 (73)	0 (0)	7 (32)	0 (0)	0 (0)	4 (18)	6 (27)
Spain Early Neolithic	6	4 (67)	5 (83)	0 (0)	6 (100)	4 (67)	4 (67)	4 (67)	5 (83)
Spain Mid Neolithic Chalcolithic	18	7 (39)	6 (33)	0 (0)	7 (39)	5 (28)	3 (17)	5 (28)	5 (28)
Sweden Mesolithic	8	8 (100)	8 (100)	0 (0)	7 (88)	4 (50)	1 (13)	6 (75)	7 (88)
Sweden Mid Neolithic	4	4 (100)	1 (25)	1 (25)	2 (50)	1 (25)	0 (0)	4 (100)	2 (50)
Turkey Neolithic	24	23 (96)	18 (75)	0 (0)	12 (50)	3 (13)	6 (25)	8 (33)	11 (46)
		76 ± 5	61 ± 5	3 ± 2	50 ± 6	21 ± 5	33 ± 7	42 ± 5	50 ± 5

EU: Europe. CSS: Complete single nucleotide polymorphism (SNP) set; PD: Principal component analysis (PCA)-derived.

**Table 2:** Accuracy of inferring hybrid individuals using the PD’s aAIMs. The six parental populations and the number of hybrid individuals generated from them are shown. Each hybrid was represented by three datasets: CSS, PD aAIMs, and a random SNP set. The mean genetic distances (*d*) between the admixture components of these datasets per population are shown. Short distances indicate high genetic similarity.

Parental Population A	Parental Population B	# Hybrids	$\bar{d\left(\mathrm{CSS},\;\mathrm{PD}\right)}$	$\bar{d\left(\mathrm{CSS},\;random\;set\right)}$	$\bar{d\left(\mathrm{PD},\;random\;set\right)}$
Britain Iron Saxon	Britain Iron Saxon	6	0.026	0.212	0.208
Britain Iron Saxon	Russia Late Chalcolithic	9	0.009	0.610	0.601
Britain Iron Saxon	Sweden Mesolithic	9	0.051	0.344	0.337
Britain Iron Saxon	Turkey Neolithic	9	0.003	0.428	0.431
Britain Iron Saxon	Spain Early Neolithic	9	0.108	0.221	0.241
Russia Late Chalcolithic	Russia Late Chalcolithic	6	0.009	0.443	0.448
Russia Late Chalcolithic	Sweden Mesolithic	9	0.062	0.578	0.561
Russia Late Chalcolithic	Turkey Neolithic	9	0.063	0.661	0.633
Russia Late Chalcolithic	Spain Early Neolithic	9	0.101	0.520	0.491
Sweden Mesolithic	Sweden Mesolithic	6	0.000	0.384	0.384
Sweden Mesolithic	Turkey Neolithic	9	0.055	0.567	0.522
Spain Early Neolithic	Sweden Mesolithic	9	0.108	0.402	0.377
Turkey Neolithic	Turkey Neolithic	6	0.001	0.627	0.626
Spain Early Neolithic	Turkey Neolithic	9	0.092	0.483	0.493
Spain Early Neolithic	Spain Early Neolithic	6	0.041	0.197	0.172

CSS: Complete single nucleotide polymorphism (SNP) set; PD: Principal component analysis (PCA)-derived.

## Supplementary Materials

The following are available online at http://www.mdpi.com/2073-4425/9/12/625/s1, Figure S1: Scatter plot of all ancient populations along the first two principal components, Figure S2: ADMIXTURE results of ancient genomes at K=2 through K=12, Figure S3: Cross-validation (CV) error and standard error of ADMIXTURE runs of K ranging from 1 to 15, Figure S4: Ancient population structure inferred by ADMIXTURE analysis, Figure S5: Minor allele frequency distributions for aAIMs identified with various methods, Figure S6: PCA plots of the aAIMs candidates identified by various methods, Figure S7: A supervised ADMIXUTRE analysis of the aAIMs candidates identified by various methods, Figure S8: A distribution of the Euclidean distances between the admixture proportions of the ancient genomes obtained from the CSS and those obtained by the aAIMs of each method, Figure S9: The effect of data missingness in predictions made using the CSS, Figure S10: Genome wide distribution of SNPs in the CSS (dots) and PD (red bars) datasets, Figure S11: Illustrating how PCA-derived (PD) aAIMs are obtained for Caucasus populations (Chalcolithic – Bronze), Table S1: Summary of aDNA samples used in this study, Table S2: Genomes retained in each method after removing samples with high missingness for the dataset of 302 genomes, Table S3: Overlapping SNPs with rs# between different methods, Table S4: Functional and frequency annotation of the studied SNPs, Additional File 1: aDNA dataset, Additional File 2: aAIM candidates produced by various tools, Additional File 3: ancient putative ancestral populations.
