# Supplementary material for: Ancient Ancestry Informative Markers for Identifying Fine-Scale Ancient Population Structure in Eurasians
Source: Genes (Basel). 2018 Dec 12;9(12):625. doi: 10.3390/genes9120625 (PMC6316245; doi:10.3390/genes9120625)
Supplement: Supplementary file 1 [file genes-09-00625-s001.zip › genes-393948-supplementary-material-final.docx]

Supplementary materials

**Figure S1.** Scatter plot of all ancient populations along the first two principal components. Symbols correspond to individuals and their color and shape correspond to the location map and the era table, respectively (right legend boxes).


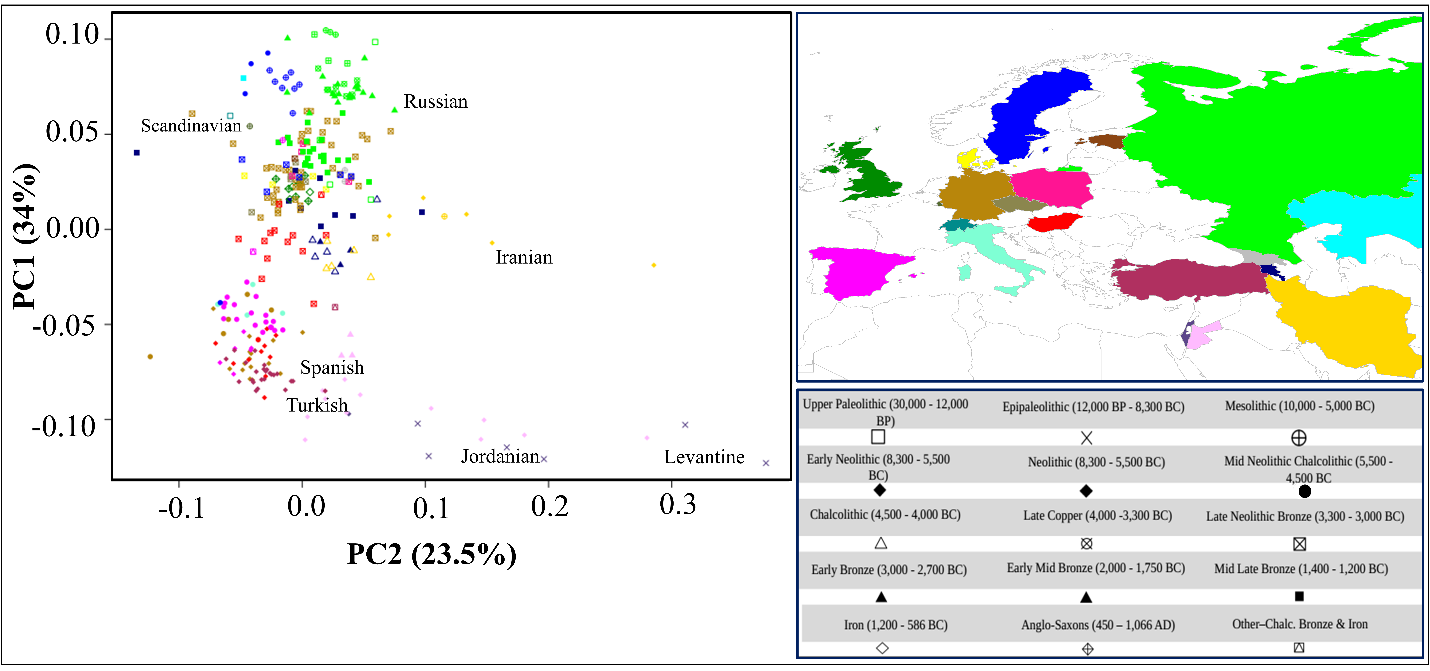


**Figure S2.** ADMIXTURE results of ancient genomes at *K*=2 through *K*=12. Each genome is represented by a vertical line partitioned into colored segments whose lengths are proportional to the contributions of the ancestral components to the genome.


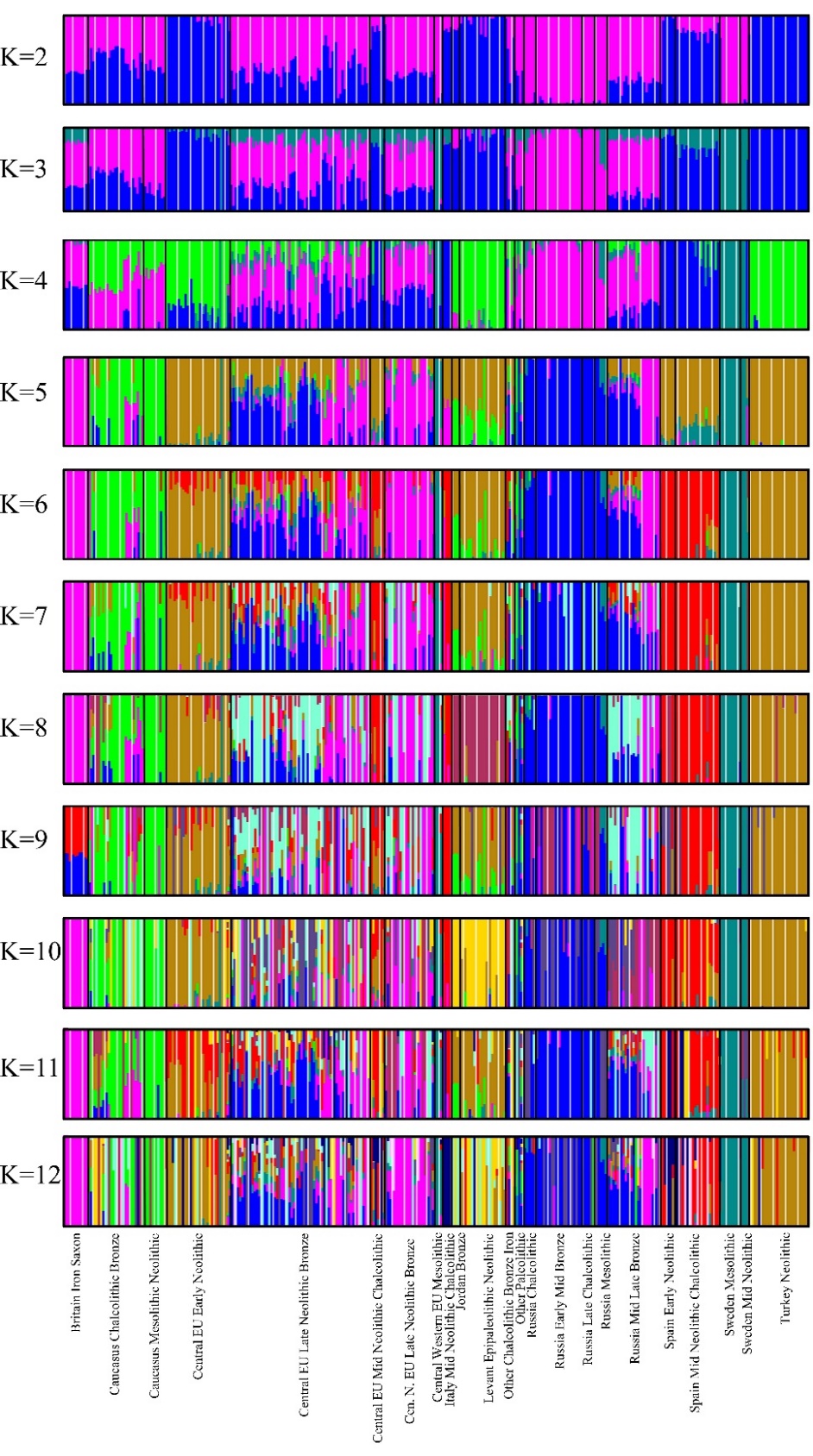


**Figure S3.** Cross-validation (CV) error and standard error of ADMIXTURE runs of *K* ranging from 1 to 15.


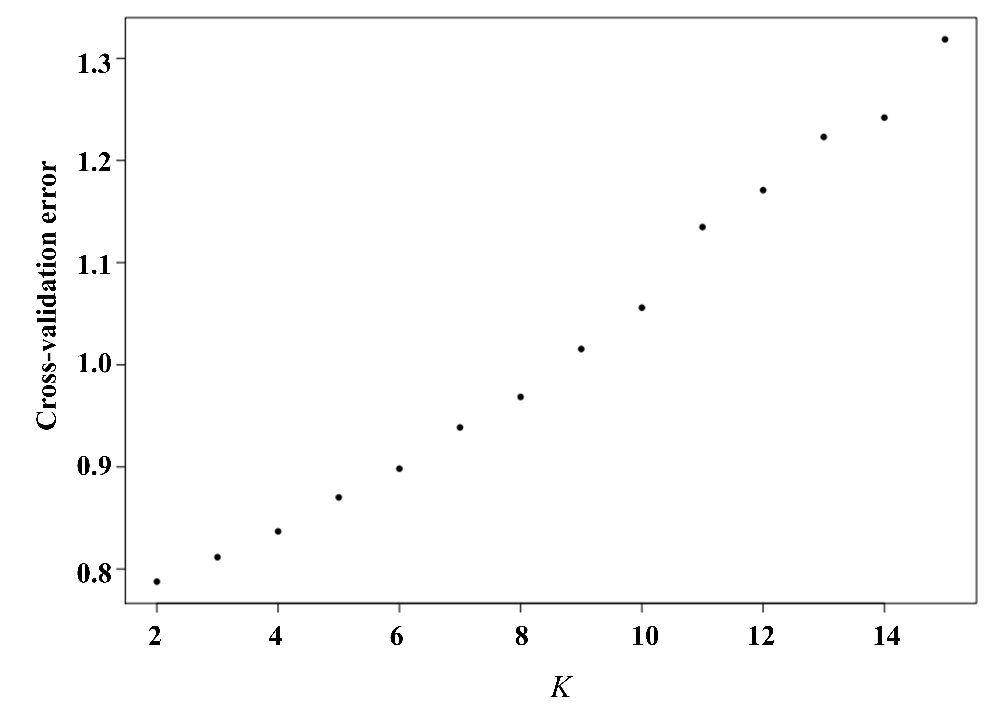


**Figure S4.** Ancient population structure inferred by ADMIXTURE analysis. Each individual is represented by a vertical (100%) stacked column of genetic components shown in color for *K*=10.


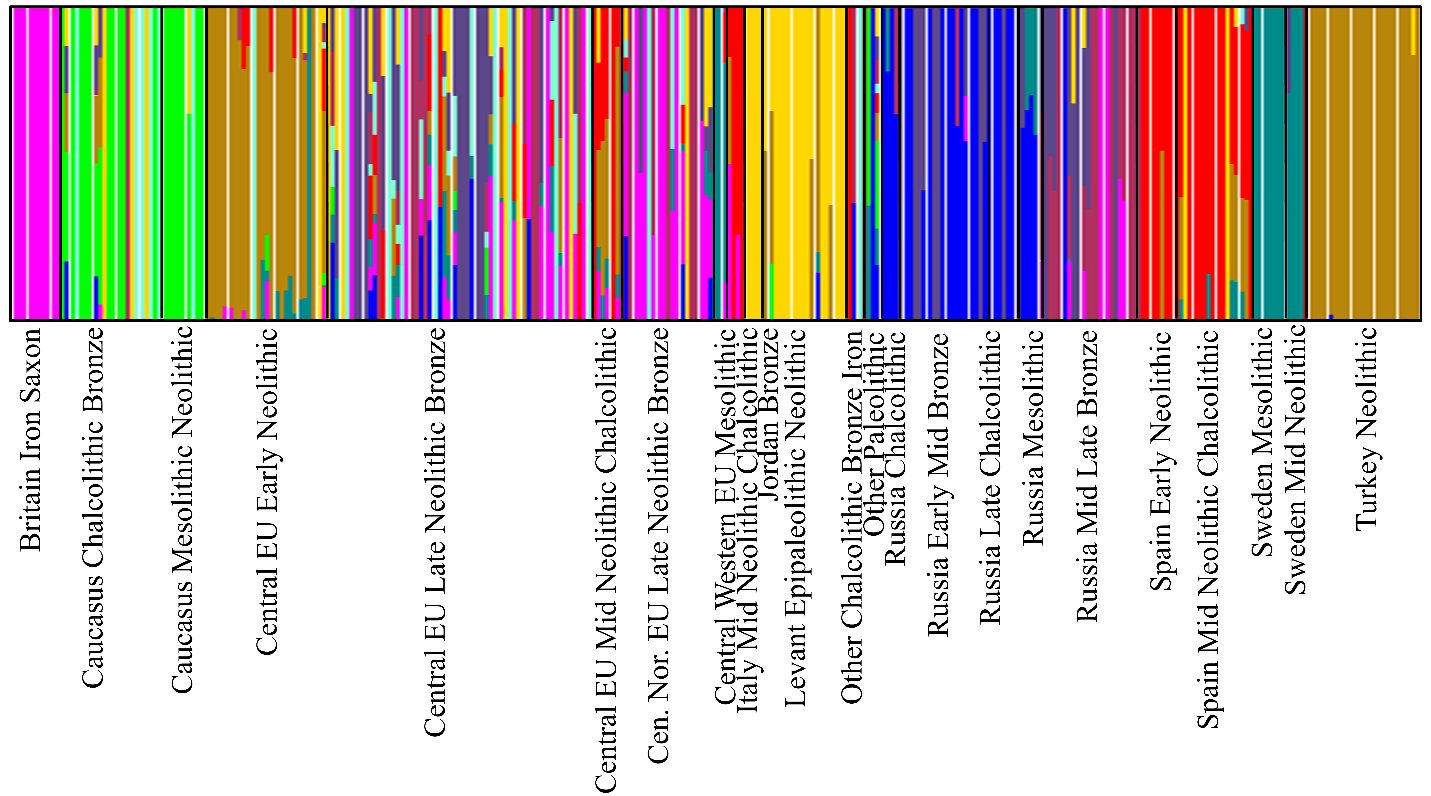


**Figure S5.** Minor allele frequency distributions for aAIMs identified with various methods. MAF frequencies were calculated for ancient (A) and modern-day (B) populations. To avoid confusion, the distributions represent the frequency of the minor allele in each dataset, which was the same one in 91.5% of the genotypes.


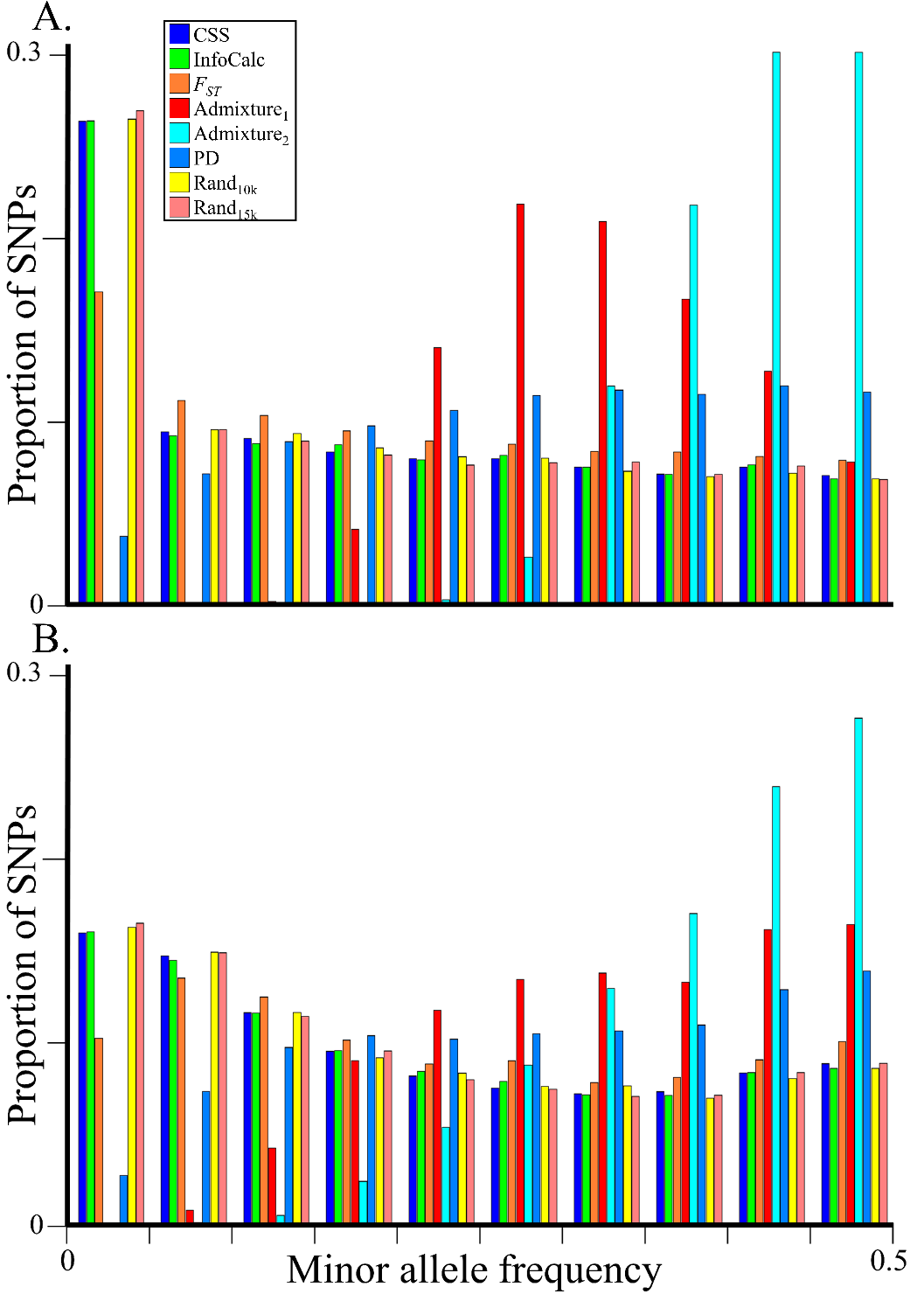


**Figure S6.** PCA plots of the aAIMs candidates identified by various methods. PCA results are shown for Infocalc (A), *F_ST_* (B), Admixture_1_ (C), Admixture_2_ (D), PD (E), Rand_10k_ (F), and Rand_15k_ (G). The color and the shape of the ancient genomes in the PCA plots correspond to the location map and the era table respectively (top right).


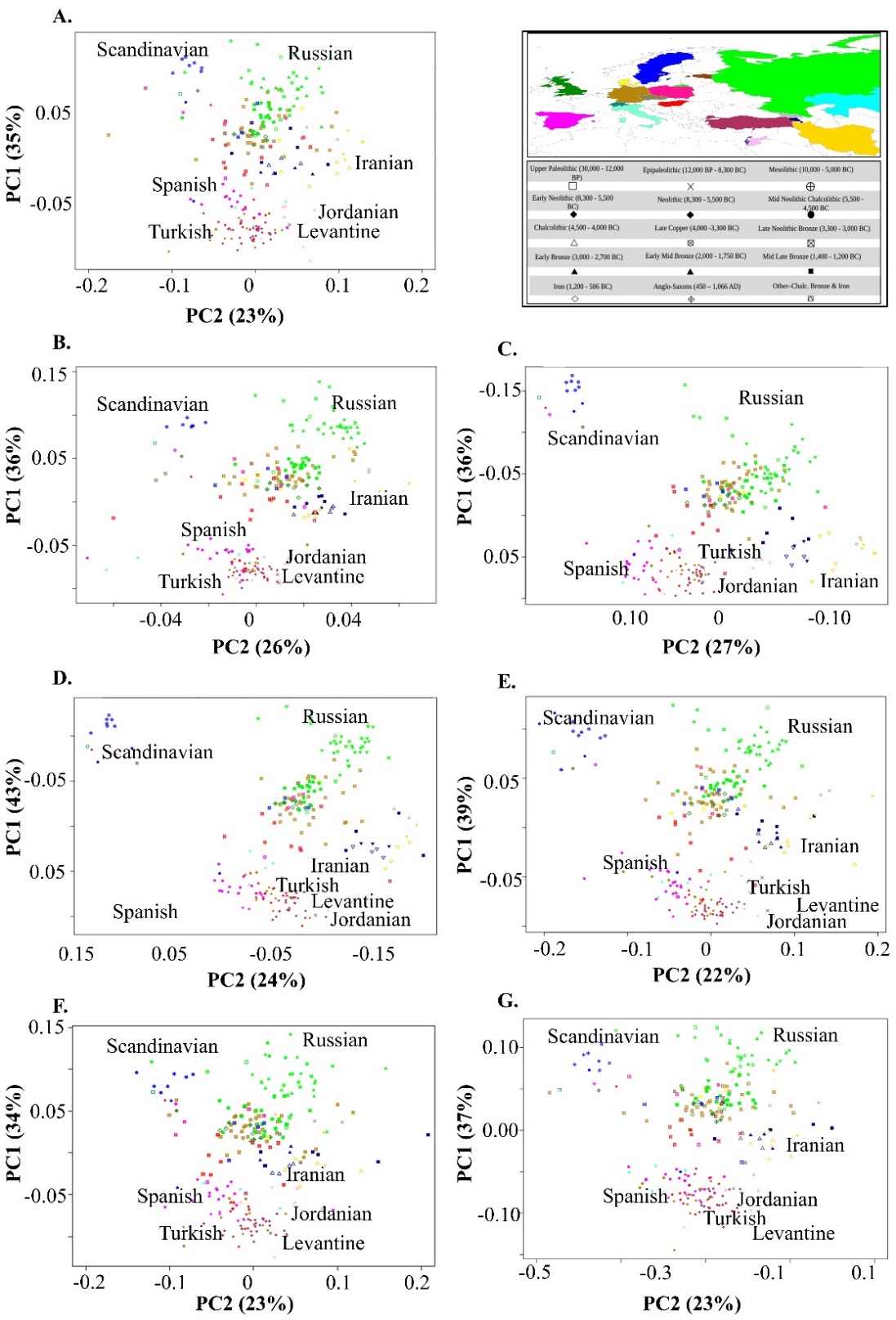


**Figure S7.** A supervised ADMIXUTRE analysis of the aAIMs candidates identified by various methods. ADMIXUTRE results are shown for Infocalc (A), *F_ST_* (B), Admixture_1_ (C), Admixture_2_ (D), PD (E), Rand_10k_ (F), and Rand_15k_ (G). Each genome is represented by a vertical line partitioned into colored segments whose lengths are proportional to the contributions of the ancestral components to the genome.


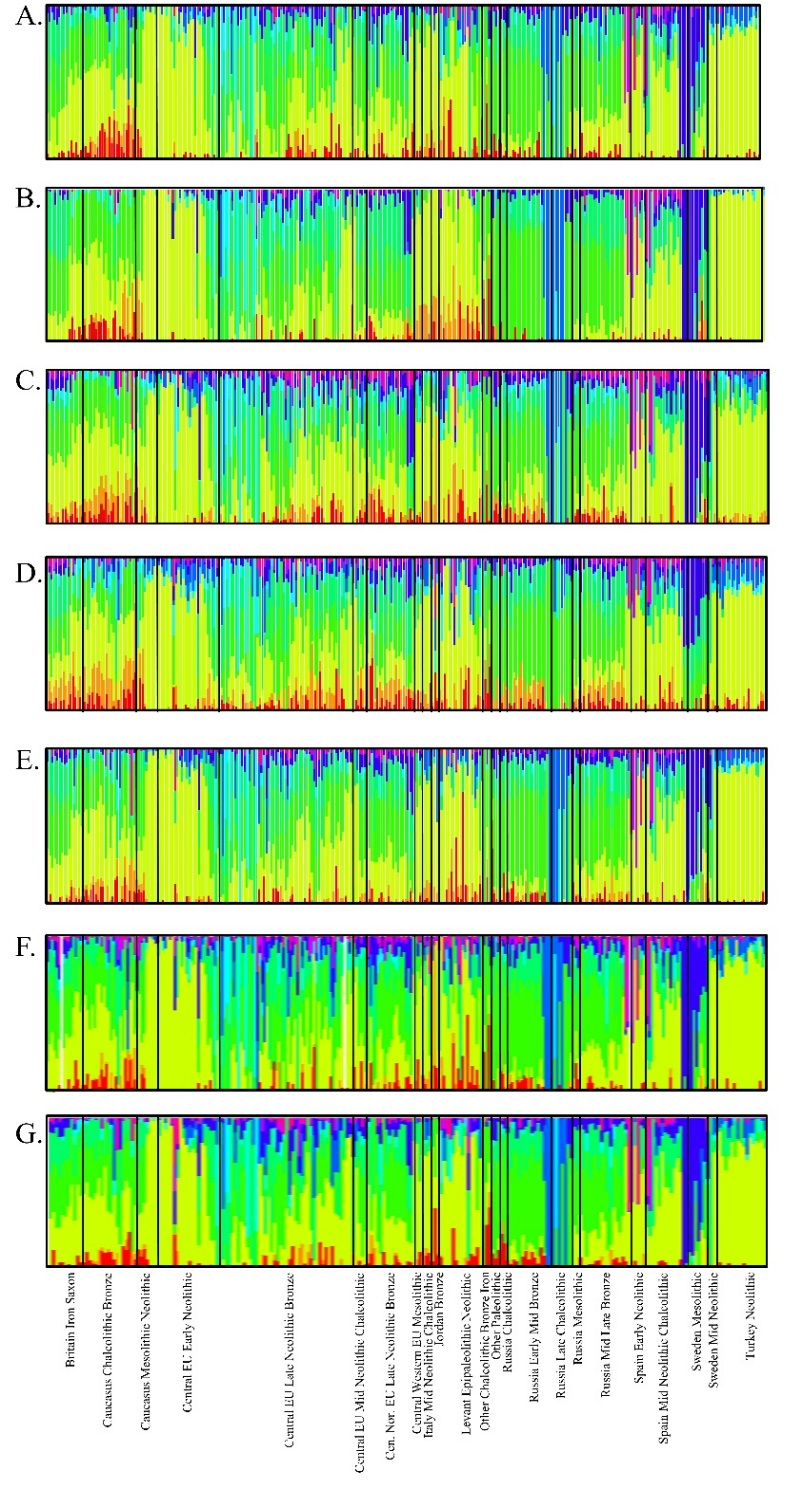


**Figure S8.** A distribution of the Euclidean distances between the admixture proportions of the ancient genomes obtained from the CSS and those obtained by the aAIMs of each method. Results are shown for Infocalc (A), *F_ST_* (B), Admixture_1_ (C), Admixture_2_ (D), PD (E), Rand_10k_ (F), and Rand_15k_ (G).


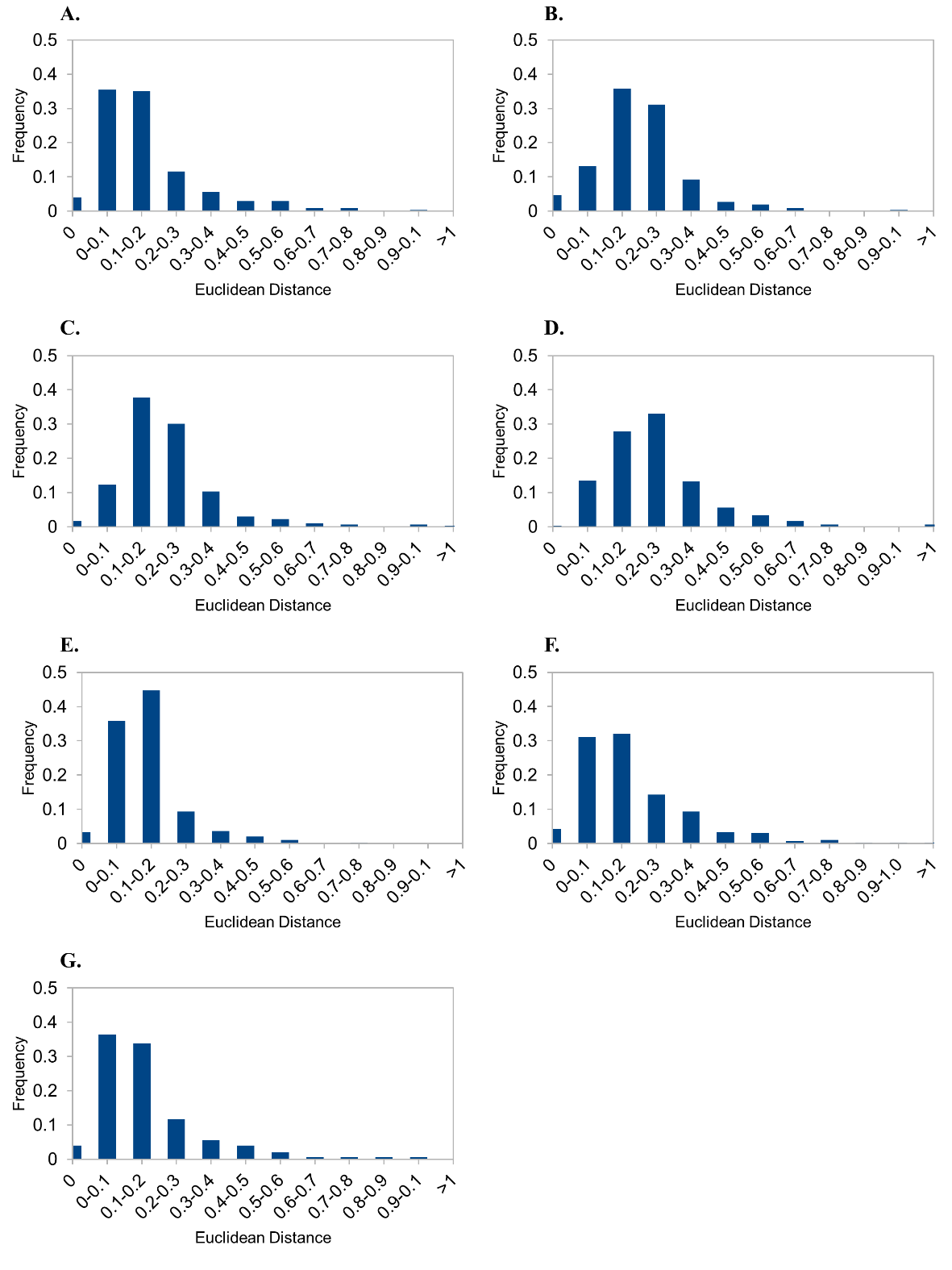


**Figure S9.** The effect of data missingness in predictions made using the CSS. Box plot shows that SNP missingness was associated with higher rate of incorrect predictions.


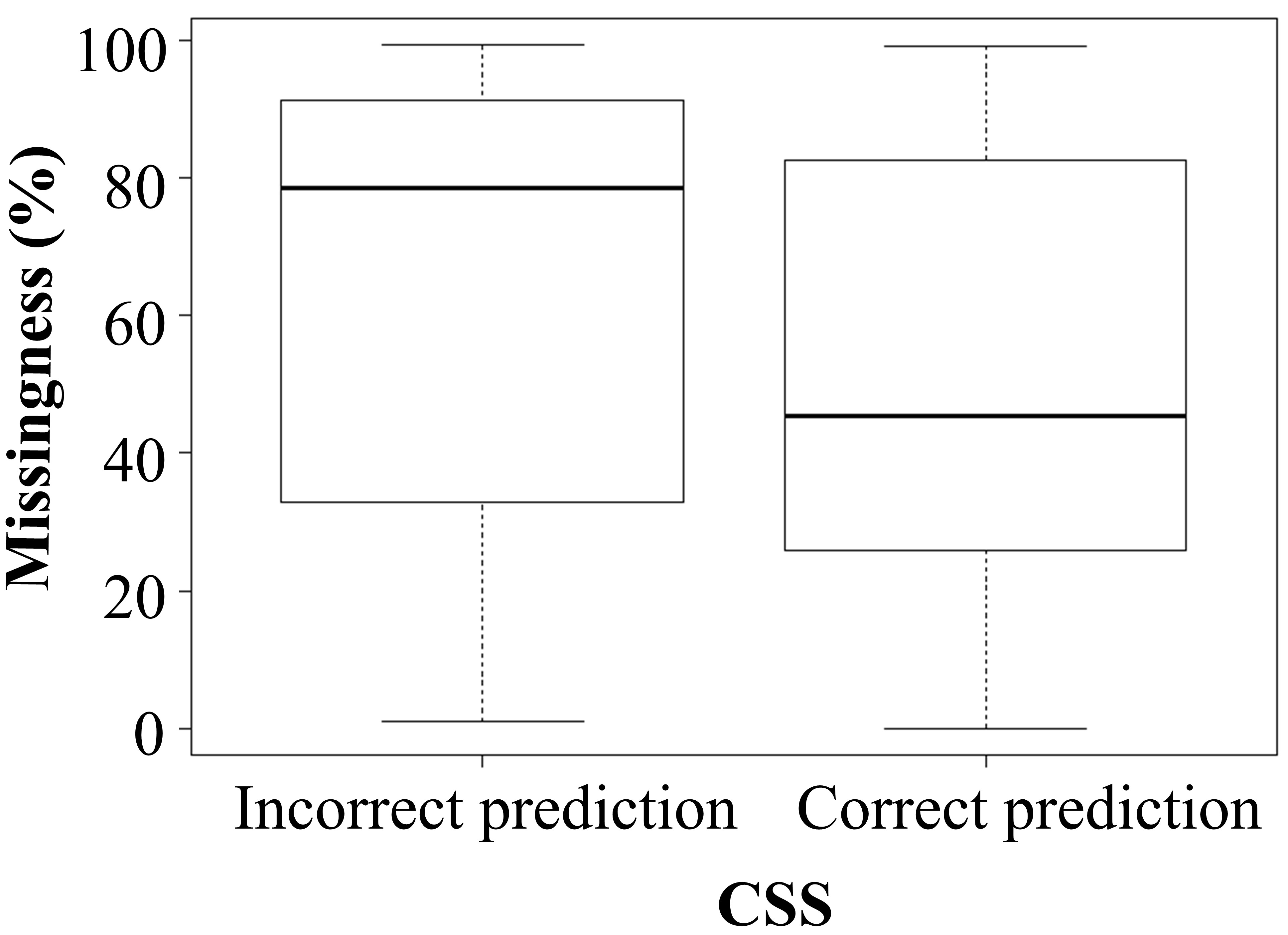


**Figure S10.** Genome wide distribution of SNPs in the CSS (dots) and PD (red bars) datasets.


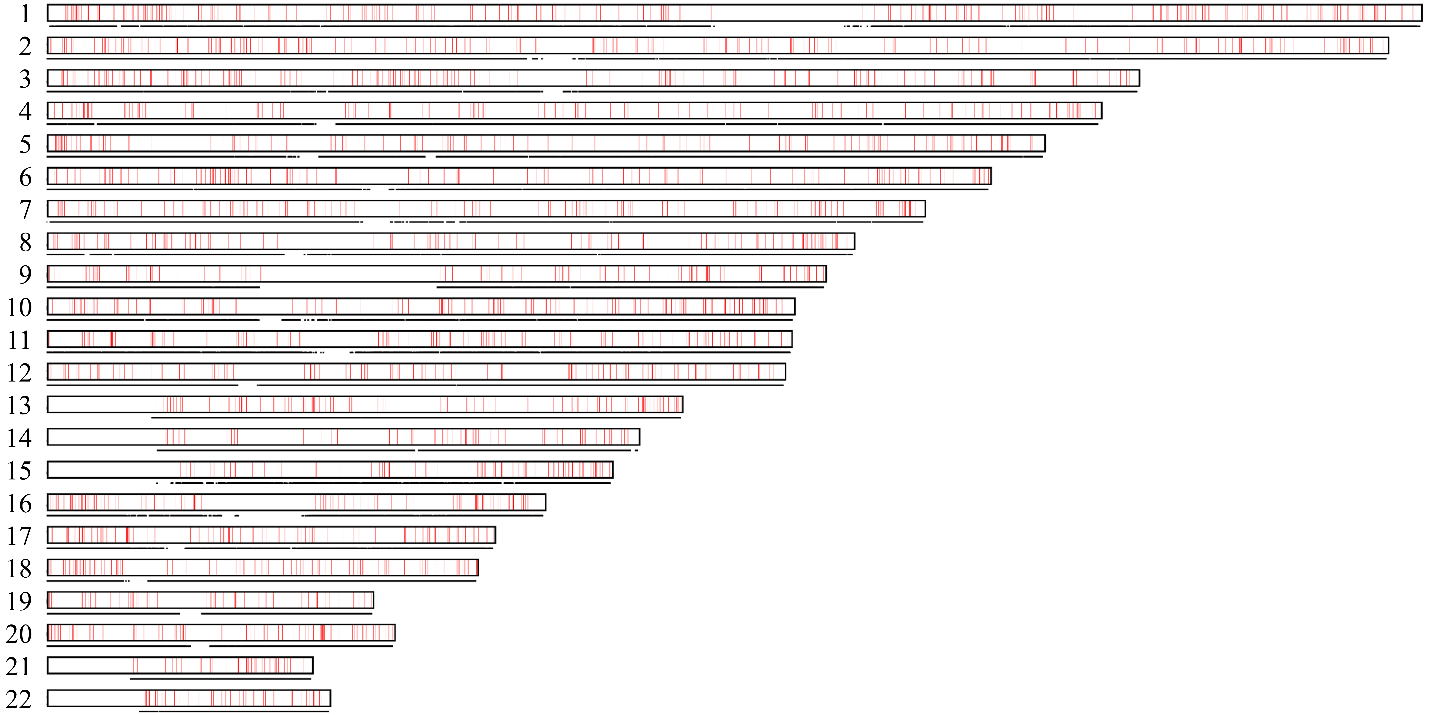


**Figure S11.** Illustrating how PCA**-**derived (PD) aAIMs are obtained for Caucasus populations **(**Chalcolithic **–** Bronze**)**. First (A), a PCA plot is drawn using the entire dataset. Next (B), the 100 first most informative markers are plotted alongside figure A and compared visually. If the B plot exhibits a different distribution it is recalculated using the 200 most informative markers (C). The process repeats until the two plots are sufficiently similar. Here, only 200 SNPs were necessary to reproduce the original distribution.


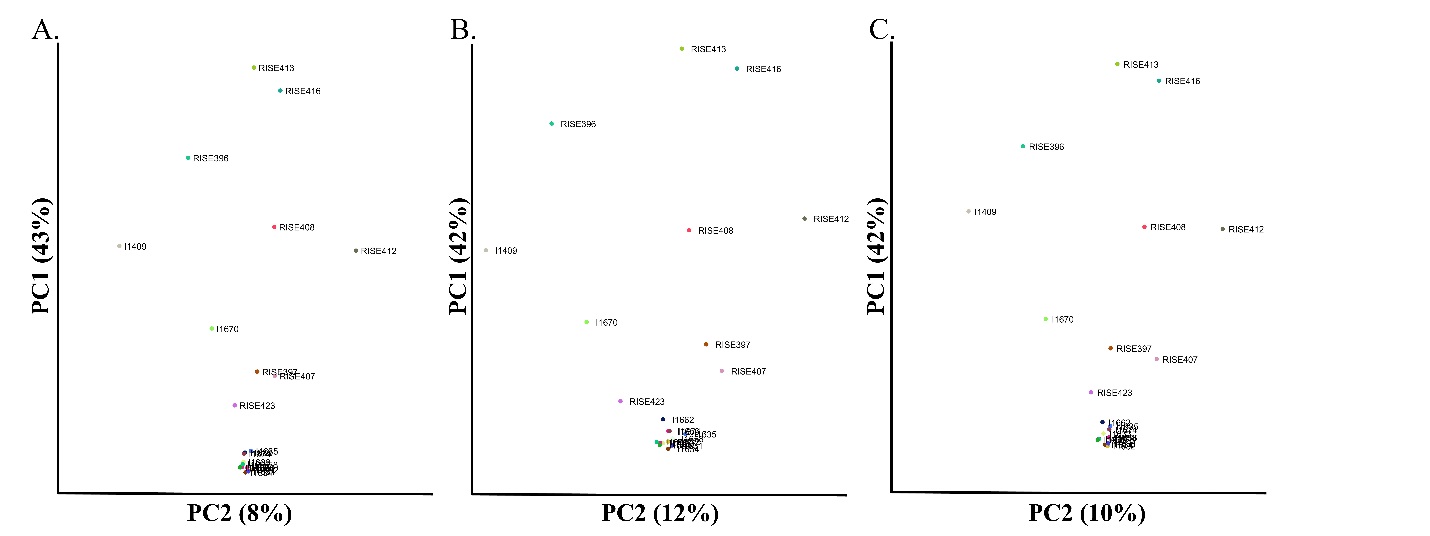


**Table S1.** Summary of aDNA samples used in this study.

| **ID** | **Alternative ID** | **Population** | **Country** | **Source** |
| --- | --- | --- | --- | --- |
| Ajvide52 | - | Sweden Mid Neolithic | Sweden | (Olalde et al. 2014) |
| Ajvide58 | - | Sweden Mid Neolithic | Sweden | (Skoglund et al. 2014) |
| Ajvide70 | - | Sweden Mid Neolithic | Sweden | (Olalde et al. 2014) |
| ATP16 | ATP16 | Spain Mid Neolithic Chalcolithic | Spain | (Lazaridis et al. 2016) |
| ATP2 | ATP2 | Spain Mid Neolithic Chalcolithic | Spain | (Lazaridis et al. 2016) |
| ATP9 | ATP9 | Other Chalcolithic Bronze Iron | Other | (Lazaridis et al. 2016) |
| Bichon | Bichon | Central Western EU Mesolithic | Central Western EU | (Lazaridis et al. 2016) |
| CB13 | CB13 | Spain Early Neolithic | Spain | (Skoglund et al. 2014) |
| Gokhem2 | - | Sweden Mid Neolithic | Sweden | (Lazaridis et al. 2016) |
| I0011 | Motala1 | Sweden Mesolithic | Sweden | (Haak et al. 2015) |
| I0012 | Motala2 | Sweden Mesolithic | Sweden | (Haak et al. 2015) |
| I0013 | Motala3 | Sweden Mesolithic | Sweden | (Haak et al. 2015) |
| I0014 | Motala4 | Sweden Mesolithic | Sweden | (Haak et al. 2015) |
| I0015 | Motala6 | Sweden Mesolithic | Sweden | (Haak et al. 2015) |
| I0016 | Motala9 | Sweden Mesolithic | Sweden | (Haak et al. 2015) |
| I0017 | Motala12 | Sweden Mesolithic | Sweden | (Haak et al. 2015) |
| I0022 | LBK1976 | Central EU Early Neolithic | Central EU | (Haak et al. 2015) |
| I0025 | LBK1992 | Central EU Early Neolithic | Central EU | (Haak et al. 2015) |
| I0026 | LBK2155 | Central EU Early Neolithic | Central EU | (Haak et al. 2015) |
| I0046 | HAL5 | Central EU Early Neolithic | Central EU | (Haak et al. 2015) |
| I0047 | HAL16 | Central EU Late Neolithic Bronze | Central EU | (Haak et al. 2015) |
| I0048 | HAL25 | Central EU Early Neolithic | Central EU | (Haak et al. 2015) |
| I0049 | ESP22 | Central EU Late Neolithic Bronze | Central EU | (Haak et al. 2015) |
| I0054 | UWS4 | Central EU Early Neolithic | Central EU | (Haak et al. 2015) |
| I0056 | HAL14 | Central EU Early Neolithic | Central EU | (Haak et al. 2015) |
| I0057 | HAL34 | Central EU Early Neolithic | Central EU | (Haak et al. 2015) |
| I0058 | BZH4 | Central EU Late Neolithic Bronze | Central EU | (Haak et al. 2015) |
| I0059 | BZH6 | Central EU Late Neolithic Bronze | Central EU | (Haak et al. 2015) |
| I0060 | ROT3 | Central EU Late Neolithic Bronze | Central EU | (Haak et al. 2015) |
| I0061 | UzOO74 | Russia Mesolithic | Russia | (Haak et al. 2015) |
| I0099 | HAL36C | Central EU Late Neolithic Bronze | Central EU | (Haak et al. 2015) |
| I0100 | HAL4 | Central EU Early Neolithic | Central EU | (Haak et al. 2015) |
| I0103 | ESP16 | Central EU Late Neolithic | Central EU | (Haak et al. 2015) |
| I0104 | ESP11 | Central EU Late Neolithic Bronze | Central EU | (Haak et al. 2015) |
| I0106 | ESP26 | Central EU Late Neolithic Bronze | Central EU | (Haak et al. 2015) |
| I0108 | ROT6 | Central EU Late Neolithic Bronze | Central EU | (Haak et al. 2015) |
| I0111 | ROT4 | Central EU Late Neolithic Bronze | Central EU | (Haak et al. 2015) |
| I0112 | QUEXII6 | Central EU Late Neolithic Bronze | Central EU | (Haak et al. 2015) |
| I0113 | QUEXII4 | Central EU Late Neolithic Bronze | Central EU | (Haak et al. 2015) |
| I0114 | ESP2 | Central EU Late Neolithic Bronze | Central EU | (Haak et al. 2015) |
| I0115 | ESP3 | Central EU Late Neolithic Bronze | Central EU | (Haak et al. 2015) |
| I0116 | ESP4 | Central EU Late Neolithic Bronze | Central EU | (Haak et al. 2015) |
| I0117 | ESP29 | Central EU Late Neolithic Bronze | Central EU | (Haak et al. 2015) |
| I0118 | ALB3 | Central EU Late Neolithic Bronze | Central EU | (Haak et al. 2015) |
| I0122 | SVP35 | Russia Chalcolithic | Russia | (Lazaridis et al. 2016) |
| I0124 | SVP44 | Russia Mesolithic | Russia | (Haak et al. 2015) |
| I0126 | SVP51 | Russia Early Mid Bronze | Russia | (Lazaridis et al. 2016) |
| I0164 | QUEVIII6 | Central EU Late Neolithic Bronze | Central EU | (Haak et al. 2015) |
| I0171 | BZH12 | Central EU Late Neolithic Bronze | Central EU | (Haak et al. 2015) |
| I0172 | ESP24 | Central EU Mid Neolithic Chalcolithic | Central EU | (Haak et al. 2015) |
| I0174 | BAM25 | Central EU Early Neolithic | Central EU | (Haak et al. 2015) |
| I0176 | SZEH4 | Central EU Early Neolithic | Central EU | (Haak et al. 2015) |
| I0211 | UzOO40 | Russia Mesolithic | Russia | (Lazaridis et al. 2016) |
| I0231 | SVP3 | Russia Late Chalcolithic | Russia | (Haak et al. 2015) |
| I0232 | SVP12 | Russia Mid Late Bronze | Russia | (Lazaridis et al. 2016) |
| I0234 | SVP25 | Russia Mid Late Bronze | Russia | (Lazaridis et al. 2016) |
| I0235 | SVP26 | Russia Mid Late Bronze | Russia | (Lazaridis et al. 2016) |
| I0246 | SVP41 | Russia Early Mid Bronze | Russia | (Lazaridis et al. 2016) |
| I0247 | SVP56 | Other Chalcolithic Bronze Iron | Other | (Lazaridis et al. 2016) |
| I0354 | SVP1 | Russia Early Mid Bronze | Russia | (Lazaridis et al. 2016) |
| I0357 | SVP5 | Russia Late Chalcolithic | Russia | (Haak et al. 2015) |
| I0358 | SVP6 | Russia Mid Late Bronze | Russia | (Lazaridis et al. 2016) |
| I0359 | SVP7 | Russia Mid Late Bronze | Russia | (Lazaridis et al. 2016) |
| I0360 | SVP8 | Russia Mid Late Bronze | Russia | (Lazaridis et al. 2016) |
| I0361 | SVP9 | Russia Mid Late Bronze | Russia | (Lazaridis et al. 2016) |
| I0370 | SVP10 | Russia Late Chalcolithic | Russia | (Haak et al. 2015) |
| I0371 | SVP11 | Russia Early Mid Bronze | Russia | (Lazaridis et al. 2016) |
| I0374 | SVP16, NIK7 | Russia Early Mid Bronze | Russia | (Lazaridis et al. 2016) |
| I0405 | Mina3 | Spain Mid Neolithic Chalcolithic | Spain | (Haak et al. 2015) |
| I0406 | Mina4 | Spain Mid Neolithic Chalcolithic | Spain | (Haak et al. 2015) |
| I0407 | Mina6 | Spain Mid Neolithic Chalcolithic | Spain | (Haak et al. 2015) |
| I0408 | Mina18 | Spain Mid Neolithic Chalcolithic | Spain | (Haak et al. 2015) |
| I0409 | Troc1 | Spain Early Neolithic | Spain | (Haak et al. 2015) |
| I0410 | Troc3 | Spain Early Neolithic | Spain | (Haak et al. 2015) |
| I0411 | Troc4 | Spain Early Neolithic | Spain | (Haak et al. 2015) |
| I0412 | Troc5 | Spain Early Neolithic | Spain | (Haak et al. 2015) |
| I0413 | Troc7 | Spain Early Neolithic | Spain | (Haak et al. 2015) |
| I0418 | SVP24 | Russia Early Mid Bronze | Russia | (Lazaridis et al. 2016) |
| I0419 | SVP27 | Russia Mid Late Bronze | Russia | (Lazaridis et al. 2016) |
| I0422 | SVP30 | Russia Mid Late Bronze | Russia | (Lazaridis et al. 2016) |
| I0423 | SVP31 | Russia Mid Late Bronze | Russia | (Lazaridis et al. 2016) |
| I0424 | SVP32 | Russia Mid Late Bronze | Russia | (Lazaridis et al. 2016) |
| I0429 | SVP38 | Russia Late Chalcolithic | Russia | (Haak et al. 2015) |
| I0430 | SVP39 | Russia Mid Late Bronze | Russia | (Lazaridis et al. 2016) |
| I0431 | SVP40 | Russia Mid Late Bronze | Russia | (Lazaridis et al. 2016) |
| I0432 | SVP42 | Russia Mid Late Bronze | Russia | (Lazaridis et al. 2016) |
| I0433 | SVP46 | Russia Chalcolithic | Russia | (Lazaridis et al. 2016) |
| I0434 | SVP47 | Russia Chalcolithic | Russia | (Lazaridis et al. 2016) |
| I0438 | SVP50 | Russia Late Chalcolithic | Russia | (Haak et al. 2015) |
| I0439 | SVP52 | Russia Late Chalcolithic | Russia | (Haak et al. 2015) |
| I0440 | SVP53 | Russia Early Mid Bronze | Russia | (Lazaridis et al. 2016) |
| I0441 | SVP54 | Russia Late Chalcolithic | Russia | (Haak et al. 2015) |
| I0443 | SVP57 | Russia Late Chalcolithic | Russia | (Haak et al. 2015) |
| I0444 | SVP58 | Russia Late Chalcolithic | Russia | (Haak et al. 2015) |
| I0550 | KAR22A | Central EU Late Neolithic Bronze | Central EU | (Haak et al. 2015) |
| I0551 | SALZ3B | Central EU Mid Neolithic Chalcolithic | Central EU | (Lazaridis et al. 2016) |
| I0559 | QLB15D | Central EU Mid Neolithic Chalcolithic | Central EU | (Haak et al. 2015) |
| I0560 | QLB18A | Central EU Mid Neolithic Chalcolithic | Central EU | (Haak et al. 2015) |
| I0585 | LB1 | Central Western EU Mesolithic | Central Western EU | (Lazaridis et al. 2016) |
| I0659 | HAL2 | Central EU Early Neolithic | Central EU | (Haak et al. 2015) |
| I0707 | BAR2 / L11-213 | Turkey Neolithic | Turkey | (Lazaridis et al. 2016) |
| I0708 | BAR6 / L11-439 | Turkey Neolithic | Turkey | (Lazaridis et al. 2016) |
| I0709 | BAR20/ M13-170 | Turkey Neolithic | Turkey | (Lazaridis et al. 2016) |
| I0723 | T1, M229 / UH | Turkey Neolithic | Turkey | (Lazaridis et al. 2016) |
| I0724 | T2 / UP | Turkey Neolithic | Turkey | (Lazaridis et al. 2016) |
| I0726 | M15, M15.2, M15.2 / UF | Turkey Neolithic | Turkey | (Lazaridis et al. 2016) |
| I0727 | M24 / UA JK 16 | Turkey Neolithic | Turkey | (Lazaridis et al. 2016) |
| I0736 | L11-216 | Turkey Neolithic | Turkey | (Lazaridis et al. 2016) |
| I0744 | M10-275 | Turkey Neolithic | Turkey | (Lazaridis et al. 2016) |
| I0745 | M11-363 | Turkey Neolithic | Turkey | (Lazaridis et al. 2016) |
| I0746 | L11-322 | Turkey Neolithic | Turkey | (Lazaridis et al. 2016) |
| I0795 | KAR6 | Central EU Early Neolithic | Central EU | (Haak et al. 2015) |
| I0797 | KAR16A | Central EU Early Neolithic | Central EU | (Lazaridis et al. 2016) |
| I0803 | EUL41 | Central EU Late Neolithic Bronze | Central EU | (Haak et al. 2015) |
| I0804 | EUL57 | Central EU Late Neolithic Bronze | Central EU | (Haak et al. 2015) |
| I0805 | QLB26 | Central EU Late Neolithic Bronze | Central EU | (Lazaridis et al. 2016) |
| I0806 | QLB28 | Central EU Late Neolithic Bronze | Central EU | (Haak et al. 2015) |
| I0807 | ESP30 | Central EU Mid Neolithic Chalcolithic | Central EU | (Haak et al. 2015) |
| I0821 | HAL24 | Central EU Early Neolithic | Central EU | (Haak et al. 2015) |
| I0861 | Nat10 | Levant Epipaleolithic Neolithic | Levant | (Lazaridis et al. 2016) |
| I0867 | Motz1 | Levant Epipaleolithic Neolithic | Levant | (Lazaridis et al. 2016) |
| I1069 | Nat5 | Levant Epipaleolithic Neolithic | Levant | (Lazaridis et al. 2016) |
| I1072 | NAT9 | Levant Epipaleolithic Neolithic | Levant | (Lazaridis et al. 2016) |
| I1096 | BAR26 / M10-76 | Turkey Neolithic | Turkey | (Lazaridis et al. 2016) |
| I1097 | BAR271 / M10-271 | Turkey Neolithic | Turkey | (Lazaridis et al. 2016) |
| I1098 | BAR99 / M10-352 | Turkey Neolithic | Turkey | (Lazaridis et al. 2016) |
| I1099 | L11-S-488 | Turkey Neolithic | Turkey | (Lazaridis et al. 2016) |
| I1100 | M11-351 | Turkey Neolithic | Turkey | (Lazaridis et al. 2016) |
| I1101 | M11-352a | Turkey Neolithic | Turkey | (Lazaridis et al. 2016) |
| I1102 | M11-354 | Turkey Neolithic | Turkey | (Lazaridis et al. 2016) |
| I1103 | M11-S-350 | Turkey Neolithic | Turkey | (Lazaridis et al. 2016) |
| I1271 | MIR1 | Spain Mid Neolithic Chalcolithic | Spain | (Lazaridis et al. 2016) |
| I1272 | MIR2 | Spain Mid Neolithic Chalcolithic | Spain | (Lazaridis et al. 2016) |
| I1276 | MIR13 | Spain Mid Neolithic Chalcolithic | Spain | (Lazaridis et al. 2016) |
| I1277 | MIR14 | Spain Mid Neolithic Chalcolithic | Spain | (Lazaridis et al. 2016) |
| I1280 | MIR17 | Spain Mid Neolithic Chalcolithic | Spain | (Lazaridis et al. 2016) |
| I1281 | MIR18 | Spain Mid Neolithic Chalcolithic | Spain | (Lazaridis et al. 2016) |
| I1282 | MIR19 | Spain Mid Neolithic Chalcolithic | Spain | (Lazaridis et al. 2016) |
| I1284 | MIR21 | Spain Mid Neolithic Chalcolithic | Spain | (Lazaridis et al. 2016) |
| I1290 | GD13A | Caucasus Mesolithic Neolithic | Caucasus | (Lazaridis et al. 2016) |
| I1293 | HotuIIIb | Caucasus Mesolithic Neolithic | Caucasus | (Lazaridis et al. 2016) |
| I1300 | MIR22 | Spain Mid Neolithic Chalcolithic | Spain | (Lazaridis et al. 2016) |
| I1303 | MIR25 | Spain Mid Neolithic Chalcolithic | Spain | (Lazaridis et al. 2016) |
| I1314 | MIR26 | Spain Mid Neolithic Chalcolithic | Spain | (Lazaridis et al. 2016) |
| I1407 | ARE12.1 | Caucasus Chalcolithic Bronze | Caucasus | (Lazaridis et al. 2016) |
| I1409 | ARE20 | Caucasus Chalcolithic Bronze | Caucasus | (Lazaridis et al. 2016) |
| I1414 | AG84/1 | Levant Epipaleolithic Neolithic | Levant | (Lazaridis et al. 2016) |
| I1415 | AG84/2 | Levant Epipaleolithic Neolithic | Levant | (Lazaridis et al. 2016) |
| I1416 | AG83/1 | Levant Epipaleolithic Neolithic | Levant | (Lazaridis et al. 2016) |
| I1495 | HUNG347, NE7 | Central EU Early Neolithic | Central EU | (Gamba et al. 2014) |
| I1496 | HUNG352, NE6 | Central EU Early Neolithic | Central EU | (Gamba et al. 2014) |
| I1497 | HUNG353, CO1 | Central EU Mid Neolithic Chalcolithic | Central EU | (Gamba et al. 2014) |
| I1498 | HUNG302, NE2 | Central EU Early Neolithic | Central EU | (Gamba et al. 2014) |
| I1499 | HUNG86, NE3 | Central EU Early Neolithic | Central EU | (Gamba et al. 2014) |
| I1500 | HUNG372, NE5 | Central EU Early Neolithic | Central EU | (Gamba et al. 2014) |
| I1502 | HUNG370, BR1 | Central EU Late Neolithic Bronze | Central EU | (Gamba et al. 2014) |
| I1504 | HUNG381, BR2 | Central EU Late Neolithic Bronze | Central EU | (Gamba et al. 2014) |
| I1505 | PF839/1198, NE4 | Central EU Early Neolithic | Central EU | (Gamba et al. 2014) |
| I1506 | PF325, NE1 | Central EU Early Neolithic | Central EU | (Gamba et al. 2014) |
| I1507 | HUNG345a, KO1 | Central EU Early Neolithic | Central EU | (Gamba et al. 2014) |
| I1508 | HUNG276, KO2 | Central EU Early Neolithic | Central EU | (Gamba et al. 2014) |
| I1532 | ESP8 | Central EU Late Neolithic Bronze | Central EU | (Lazaridis et al. 2016) |
| I1534 | ESP14 | Central EU Late Neolithic Bronze | Central EU | (Lazaridis et al. 2016) |
| I1536 | ESP17 | Central EU Late Neolithic Bronze | Central EU | (Lazaridis et al. 2016) |
| I1538 | ESP20 | Central EU Late Neolithic Bronze | Central EU | (Lazaridis et al. 2016) |
| I1539 | ESP25 | Central EU Late Neolithic Bronze | Central EU | (Lazaridis et al. 2016) |
| I1540 | ESP28 | Central EU Late Neolithic Bronze | Central EU | (Lazaridis et al. 2016) |
| I1542 | ESP33 | Central EU Late Neolithic Bronze | Central EU | (Lazaridis et al. 2016) |
| I1544 | ESP36 | Central EU Late Neolithic Bronze | Central EU | (Lazaridis et al. 2016) |
| I1546 | BZH2 | Central EU Late Neolithic Bronze | Central EU | (Lazaridis et al. 2016) |
| I1549 | BZH15 | Central EU Late Neolithic Bronze | Central EU | (Lazaridis et al. 2016) |
| I1550 | HAL19 | Central EU Early Neolithic | Central EU | (Lazaridis et al. 2016) |
| I1579 | M13-72 | Turkey Neolithic | Turkey | (Lazaridis et al. 2016) |
| I1580 | L12-393 | Turkey Neolithic | Turkey | (Lazaridis et al. 2016) |
| I1581 | L12-502 | Turkey Neolithic | Turkey | (Lazaridis et al. 2016) |
| I1583 | L14-200 | Turkey Neolithic | Turkey | (Lazaridis et al. 2016) |
| I1584 | M10-111 | Other Chalcolithic Bronze Iron | Other | (Lazaridis et al. 2016) |
| I1585 | M11-59 | Turkey Neolithic | Turkey | (Lazaridis et al. 2016) |
| I1631 | AR1/43C | Caucasus Chalcolithic Bronze | Caucasus | (Lazaridis et al. 2016) |
| I1632 | AR1/46 | Caucasus Chalcolithic Bronze | Caucasus | (Lazaridis et al. 2016) |
| I1633 | KA1/14 | Caucasus Chalcolithic Bronze | Caucasus | (Lazaridis et al. 2016) |
| I1634 | AR1/44 | Caucasus Chalcolithic Bronze | Caucasus | (Lazaridis et al. 2016) |
| I1635 | KA1/12 | Caucasus Chalcolithic Bronze | Caucasus | (Lazaridis et al. 2016) |
| I1656 | Kat16 | Caucasus Chalcolithic Bronze | Caucasus | (Lazaridis et al. 2016) |
| I1658 | TA3/R8 | Caucasus Chalcolithic Bronze | Caucasus | (Lazaridis et al. 2016) |
| I1661 | SG16 | Caucasus Chalcolithic Bronze | Caucasus | (Lazaridis et al. 2016) |
| I1662 | SG7 | Caucasus Chalcolithic Bronze | Caucasus | (Lazaridis et al. 2016) |
| I1665 | SG19 | Caucasus Chalcolithic Bronze | Caucasus | (Lazaridis et al. 2016) |
| I1670 | SG11 | Caucasus Chalcolithic Bronze | Caucasus | (Lazaridis et al. 2016) |
| I1671 | SG2 | Caucasus Mesolithic Neolithic | Caucasus | (Lazaridis et al. 2016) |
| I1674 | SG21 | Caucasus Chalcolithic Bronze | Caucasus | (Lazaridis et al. 2016) |
| I1679 | AG037C | Levant Epipaleolithic Neolithic | Levant | (Lazaridis et al. 2016) |
| I1685 | NAT4 | Levant Epipaleolithic Neolithic | Levant | (Lazaridis et al. 2016) |
| I1687 | NAT13 | Levant Epipaleolithic Neolithic | Levant | (Lazaridis et al. 2016) |
| I1690 | NAT6 | Levant Epipaleolithic Neolithic | Levant | (Lazaridis et al. 2016) |
| I1699 | AG84_5 | Levant Epipaleolithic Neolithic | Levant | (Lazaridis et al. 2016) |
| I1700 | AG88_1 | Levant Epipaleolithic Neolithic | Levant | (Lazaridis et al. 2016) |
| I1701 | AG83_3 | Levant Epipaleolithic Neolithic | Levant | (Lazaridis et al. 2016) |
| I1704 | AG89_1 | Levant Epipaleolithic Neolithic | Levant | (Lazaridis et al. 2016) |
| I1705 | AG98_1 | Jordan Bronze | Jordan | (Lazaridis et al. 2016) |
| I1706 | AG98_2 | Jordan Bronze | Jordan | (Lazaridis et al. 2016) |
| I1707 | AG83_5 | Levant Epipaleolithic Neolithic | Levant | (Lazaridis et al. 2016) |
| I1709 | AG84_8 | Levant Epipaleolithic Neolithic | Levant | (Lazaridis et al. 2016) |
| I1710 | AG83_6 | Levant Epipaleolithic Neolithic | Levant | (Lazaridis et al. 2016) |
| I1727 | AG_83_3082 | Levant Epipaleolithic Neolithic | Levant | (Lazaridis et al. 2016) |
| I1730 | AG_84_3083_116 | Jordan Bronze | Jordan | (Lazaridis et al. 2016) |
| I1944 | GD14B | Caucasus Mesolithic Neolithic | Caucasus | (Lazaridis et al. 2016) |
| I1945 | GD16 | Caucasus Mesolithic Neolithic | Caucasus | (Lazaridis et al. 2016) |
| I1949 | GD37 | Caucasus Mesolithic Neolithic | Caucasus | (Lazaridis et al. 2016) |
| I1951 | GD39 | Caucasus Mesolithic Neolithic | Caucasus | (Lazaridis et al. 2016) |
| Iceman | - | Italy Mid Neolithic Chalcolithic | Italy | (Keller et al. 2012) |
| IR1 | - | Other Chalcolithic Bronze Iron | Other | (Gamba et al. 2014) |
| Iron_12880 | HI1 | Britain Iron Saxon | Britain | (Schiffels et al. 2016) |
| Iron_12884 | HI2 | Britain Iron Saxon | Britain | (Schiffels et al. 2016) |
| Iron_15579 | L | Britain Iron Saxon | Britain | (Schiffels et al. 2016) |
| KK1 | Kotias | Caucasus Mesolithic Neolithic | Caucasus | (Lazaridis et al. 2016) |
| Kostenki14 | Kostenki14 | Other Palaeol | Other | (Seguin-Orlando et al. 2014) |
| Loschbour | Loschbour | Central Western EU Mesolithic | Central Western EU | (Lazaridis et al. 2014) |
| MA1 | Malta1 | Other Palaeol | Other | (Raghavan et al. 2014) |
| Matojo | Matojo | Spain Mid Neolithic Chalcolithic | Spain | (Lazaridis et al. 2016) |
| Modern_12881 | HS1 | Britain Iron Saxon | Britain | (Schiffels et al. 2016) |
| Modern_12883 | HS2 | Britain Iron Saxon | Britain | (Schiffels et al. 2016) |
| Modern_12885 | HS3 | Britain Iron Saxon | Britain | (Schiffels et al. 2016) |
| Modern_15558 | O1 | Britain Iron Saxon | Britain | (Schiffels et al. 2016) |
| Modern_15569 | O2 | Britain Iron Saxon | Britain | (Schiffels et al. 2016) |
| Modern_15570 | O3 | Britain Iron Saxon | Britain | (Schiffels et al. 2016) |
| Modern_15577 | O4 | Britain Iron Saxon | Britain | (Schiffels et al. 2016) |
| RISE00 | grave | Central Northern EU Late Neolithic Bronze | Central Northern EU | (Lazaridis et al. 2016) |
| RISE109 | grave 1044 | Central Northern EU Late Neolithic Bronze | Central Northern EU | (Lazaridis et al. 2016) |
| RISE150 | grave 02 | Central Northern EU Late Neolithic Bronze | Central Northern EU | (Lazaridis et al. 2016) |
| RISE154 | grave 3 | Central Northern EU Late Neolithic Bronze | Central Northern EU | (Lazaridis et al. 2016) |
| RISE175 | barrow I grave 14:1 | Central Northern EU Late Neolithic Bronze | Central Northern EU | (Lazaridis et al. 2016) |
| RISE179 | barrow I grave 5:1, gallery grave | Central Northern EU Late Neolithic Bronze | Central Northern EU | (Lazaridis et al. 2016) |
| RISE210 | Cranium VI | Central Northern EU Late Neolithic Bronze | Central Northern EU | (Lazaridis et al. 2016) |
| RISE240 | kurgan 1, grave 11 | Russia Early Mid Bronze | Russia | (Lazaridis et al. 2016) |
| RISE247 | ID 3437 | Central EU Late Neolithic Bronze | Central EU | (Lazaridis et al. 2016) |
| RISE254 | ID 4091 | Central EU Late Neolithic Bronze | Central EU | (Lazaridis et al. 2016) |
| RISE276 | bog find 1940 | Central Northern EU Late Neolithic Bronze | Central Northern EU | (Lazaridis et al. 2016) |
| RISE349 | Grave # 33 | Central EU Late Neolithic Bronze | Central EU | (Lazaridis et al. 2016) |
| RISE371 | Grave # 105 | Central EU Late Neolithic Bronze | Central EU | (Lazaridis et al. 2016) |
| RISE373 | Grave # 123 | Central EU Late Neolithic Bronze | Central EU | (Lazaridis et al. 2016) |
| RISE374 | Grave # 147 | Central EU Late Neolithic Bronze | Central EU | (Lazaridis et al. 2016) |
| RISE386 | burial 4 | Russia Mid Late Bronze | Russia | (Lazaridis et al. 2016) |
| RISE391 | kurgan 7 burial 36 | Russia Mid Late Bronze | Russia | (Lazaridis et al. 2016) |
| RISE392 | kurgan 4 burial B | Russia Mid Late Bronze | Russia | (Lazaridis et al. 2016) |
| RISE394 | burial 6 skeleton 1 | Russia Mid Late Bronze | Russia | (Lazaridis et al. 2016) |
| RISE395 | kurgan 25 burial 12 | Russia Mid Late Bronze | Russia | (Lazaridis et al. 2016) |
| RISE396 | tomb 6 skeleton 1 | Caucasus Chalcolithic Bronze | Caucasus | (Lazaridis et al. 2016) |
| RISE397 | tomb 6 skeleton 2 | Caucasus Chalcolithic Bronze | Caucasus | (Lazaridis et al. 2016) |
| RISE407 | #5 | Caucasus Chalcolithic Bronze | Caucasus | (Lazaridis et al. 2016) |
| RISE408 | #6 | Caucasus Chalcolithic Bronze | Caucasus | (Lazaridis et al. 2016) |
| RISE412 | #10 | Caucasus Chalcolithic Bronze | Caucasus | (Lazaridis et al. 2016) |
| RISE413 | #11 | Caucasus Chalcolithic Bronze | Caucasus | (Lazaridis et al. 2016) |
| RISE416 | #14 | Caucasus Chalcolithic Bronze | Caucasus | (Lazaridis et al. 2016) |
| RISE423 | #21 | Caucasus Chalcolithic Bronze | Caucasus | (Lazaridis et al. 2016) |
| RISE431 | Barrow 4, skeleton 2 | Central Northern EU Late Neolithic Bronze | Central Northern EU | (Lazaridis et al. 2016) |
| RISE434 | 3/1 | Central EU Late Neolithic Bronze | Central EU | (Lazaridis et al. 2016) |
| RISE435 | 3/2 | Central EU Late Neolithic Bronze | Central EU | (Lazaridis et al. 2016) |
| RISE436 | 3/3 | Central EU Late Neolithic Bronze | Central EU | (Lazaridis et al. 2016) |
| RISE446 | burial 13 male | Central EU Late Neolithic Bronze | Central EU | (Lazaridis et al. 2016) |
| RISE47 | N 358 grave 3 skeleton 8 | Central Northern EU Late Neolithic Bronze | Central Northern EU | (Lazaridis et al. 2016) |
| RISE471 | burial 1 | Central EU Late Neolithic Bronze | Central EU | (Lazaridis et al. 2016) |
| RISE479 | ID 1129/1706 Q3 (P23) | Central EU Late Neolithic Bronze | Central EU | (Lazaridis et al. 2016) |
| RISE480 | ID 1039/1550 Q1 (P24) | Central EU Late Neolithic Bronze | Central EU | (Lazaridis et al. 2016) |
| RISE483 | ID 106/159 Q2 (P27) | Central EU Late Neolithic Bronze | Central EU | (Lazaridis et al. 2016) |
| RISE484 | ID 772/1170 Q3 (P28) | Central EU Late Neolithic Bronze | Central EU | (Lazaridis et al. 2016) |
| RISE486 | T78 | Italy Mid Neolithic Chalcolithic | Italy | (Lazaridis et al. 2016) |
| RISE487 | T56 | Italy Mid Neolithic Chalcolithic | Italy | (Lazaridis et al. 2016) |
| RISE489 | T65 | Italy Mid Neolithic Chalcolithic | Italy | (Lazaridis et al. 2016) |
| RISE500 | 6652-38 | Russia Mid Late Bronze | Russia | (Lazaridis et al. 2016) |
| RISE503 | 6652-41 | Russia Mid Late Bronze | Russia | (Lazaridis et al. 2016) |
| RISE505 | 6652-42 | Russia Mid Late Bronze | Russia | (Lazaridis et al. 2016) |
| RISE507 | 5910-2A | Russia Early Mid Bronze | Russia | (Lazaridis et al. 2016) |
| RISE508 | 5910-2B | Russia Early Mid Bronze | Russia | (Lazaridis et al. 2016) |
| RISE509 | 6136-5 | Russia Early Mid Bronze | Russia | (Lazaridis et al. 2016) |
| RISE510 | 6136-9 | Russia Early Mid Bronze | Russia | (Lazaridis et al. 2016) |
| RISE511 | 6136-6 | Russia Early Mid Bronze | Russia | (Lazaridis et al. 2016) |
| RISE546 | Kurgan 1, grave 13 | Russia Early Mid Bronze | Russia | (Lazaridis et al. 2016) |
| RISE547 | Kurgan 1, grave 9 | Russia Early Mid Bronze | Russia | (Lazaridis et al. 2016) |
| RISE548 | Kurgan 1, grave 6 | Russia Early Mid Bronze | Russia | (Lazaridis et al. 2016) |
| RISE550 | Kurgan 1, grave 3 | Russia Early Mid Bronze | Russia | (Lazaridis et al. 2016) |
| RISE552 | Kurgan 4, grave 8 | Russia Early Mid Bronze | Russia | (Lazaridis et al. 2016) |
| RISE555 | CGG_2_011887 | Russia Early Mid Bronze | Russia | (Lazaridis et al. 2016) |
| RISE559 | F0174, gr 4 | Central EU Late Neolithic Bronze | Central EU | (Lazaridis et al. 2016) |
| RISE560 | F0187, gr 3 | Central EU Late Neolithic Bronze | Central EU | (Lazaridis et al. 2016) |
| RISE562 | F0228, obj. 136/92 = gr.9 | Central EU Late Neolithic Bronze | Central EU | (Lazaridis et al. 2016) |
| RISE563 | F0234, obj. 8 | Central EU Late Neolithic Bronze | Central EU | (Lazaridis et al. 2016) |
| RISE564 | F0241, obj. 25 | Central EU Late Neolithic Bronze | Central EU | (Lazaridis et al. 2016) |
| RISE566 | F0521, A01168, gr. 14 | Central Northern EU Late Neolithic Bronze | Central Northern EU | (Lazaridis et al. 2016) |
| RISE568 | F0525, A01623, gr. 16 | Central Northern EU Late Neolithic Bronze | Central Northern EU | (Lazaridis et al. 2016) |
| RISE569 | F0527, A01643, gr. 35? | Central Northern EU Late Neolithic Bronze | Central Northern EU | (Lazaridis et al. 2016) |
| RISE577 | F0565, gr. 238 | Central Northern EU Late Neolithic Bronze | Central Northern EU | (Lazaridis et al. 2016) |
| RISE586 | F0597, gr. 6 | Central Northern EU Late Neolithic Bronze | Central Northern EU | (Lazaridis et al. 2016) |
| RISE61 | PMD 17, V, N chamber lower layer | Central Northern EU Late Neolithic Bronze | Central Northern EU | (Lazaridis et al. 2016) |
| RISE71 | PMD 57, I | Central Northern EU Late Neolithic Bronze | Central Northern EU | (Lazaridis et al. 2016) |
| RISE94 | grave 26:I | Central Northern EU Late Neolithic Bronze | Central Northern EU | (Lazaridis et al. 2016) |
| RISE97 | grave 72(II) | Central Northern EU Late Neolithic Bronze | Central Northern EU | (Lazaridis et al. 2016) |
| RISE98 | grave 49, S skeleton | Central Northern EU Late Neolithic Bronze | Central Northern EU | (Lazaridis et al. 2016) |
| SATP | Satsurblia | Caucasus Mesolithic Neolithic | Caucasus | (Lazaridis et al. 2016) |
| StoraForvar11 | - | Sweden Mesolithic | Sweden | (Skoglund et al. 2014) |
| Stuttgart | Stuttgart | Central EU Early Neolithic | Central EU | (Lazaridis et al. 2014) |
| Ust_Ishim | UstIshim | Other Palaeol | Other | (Fu et al. 2014) |

**Table S2.** Genomes retained in each method after removing samples with high missingness for the dataset of 302 genomes.

| **Method** | **Retained** | **Removed** |
| --- | --- | --- |
| Infocalc | 245 | 57 |
| *F_ST_* | 256 | 46 |
| Admixture_1_ | 248 | 54 |
| Admixture_2_ | 223 | 79 |
| PD | 263 | 39 |
| Rand_10k_ | 245 | 57 |
| Rand_15k_ | 243 | 59 |

**Table S3.** Overlapping SNPs with rs# between different methods. The number of aAIMs candidates identified by each method is shown on the diagonal overlapping SNPs. The numbers of overlapping and nonoverlapping SNPs are shown above and below the diagonal, respectively. A small number of SNPs without rs# were not analyzes.

|  | **Infocalc** | ***F_ST_*** | **Admixture_1_** | **Admixture_2_** | **PD** | **Rand_10k_** | **Rand_15k_** |
| --- | --- | --- | --- | --- | --- | --- | --- |
| Infocalc | 14,727 | 1,484 | 913 | 1,188 | 1,322 | 987 | 1,473 |
| *F_ST_* | 13,216 | 14,700 | 1,206 | 1,566 | 1,785 | 915 | 1,461 |
| Admixture_1_ | 8,373 | 8,080 | 9,286 | 2,160 | 1,333 | 560 | 862 |
| Admixture_2_ | 10,201 | 9,823 | 9,229 | 11,389 | 978 | 723 | 1,183 |
| PD | 11,650 | 11,187 | 11,639 | 11,994 | 12,972 | 805 | 1,219 |
| Rand_10k_ | 8,537 | 8,609 | 8,964 | 8,801 | 8,719 | 9,524 | 1,000 |
| Rand_15k_ | 13,262 | 13,274 | 13,873 | 13,552 | 13,516 | 13,735 | 14,735 |

**Table S4.** Functional and frequency annotation of the studied SNPs.

|  |  | **CSS** | **Infocalc** | **FST** | **Admixture1** | **Admixture2** | **PD** | **Rand10k** | **Rand15k** |
| --- | --- | --- | --- | --- | --- | --- | --- | --- | --- |
| Non-functional variants | Intron/intergenic | 74.5% | 82.4% | 81.2% | 81.6% | 83.9% | 79.5% | 81.7% | 82.0% |
|  | Down/upstream | 19.1% | 12.1% | 12.2% | 12.4% | 11.4% | 13.2% | 12.6% | 12.2% |
|  | Splice sites | 0% | 0% | 0% | 0% | 0% | 0% | 0% | 0% |
|  | UTR | 0.9% | 1.0% | 1.0% | 1.0% | 0.9% | 1.0% | 1.1% | 1.0% |
|  | Other | 1.1% | 0.6% | 0.9% | 0.8% | 0.6% | 0.9% | 0.9% | 0.7% |
| Putatively functional variants | Missense | 0.6% | 0.4% | 0.7% | 0.5% | 0.4% | 0.8% | 0.4% | 0.5% |
|  | Synonymous | 0.6% | 0.5% | 0.6% | 0.5% | 0.3% | 0.8% | 0.5% | 0.5% |
|  | Start/Stop | 0% | 0% | 0% | 0% | 0% | 0% | 0% | 0% |
|  | Regulatory | 3.2% | 2.9% | 3.5% | 3.3% | 2.5% | 3.7% | 2.8% | 3.0% |
|  | Coding | 0% | 0% | 0% | 0% | 0% | 0% | 0% | 0% |
|  | Missense variant | 0.6% | 0.4% | 0.7% | 0.5% | 0.4% | 0.8% | 0.4% | 0.5% |

**References**

Fu, Q.; Li, H.; Moorjani, P.; Jay, F.; Slepchenko, S. M.; Bondarev, A. A.; Johnson, P. L. F.; Aximu-Petri, A.; Prüfer, K.; de Filippo, C.; et al. Genome sequence of a 45,000-year-old modern human from western Siberia. *Nature* **2014**, *514*, 445-449.

Gamba, C.; Jones, E. R.; Teasdale, M. D.; McLaughlin, R. L.; Gonzalez-Fortes, G.; Mattiangeli, V.; Domboróczki, L.; Kővári, I.; Pap, I.; Anders, A.; et al. Genome flux and stasis in a five millennium transect of European prehistory. *Nat. Commun.* **2014**, *5*, 5257.

Haak, W.; Lazaridis, I.; Patterson, N.; Rohland, N.; Mallick, S.; Llamas, B.; Brandt, G.; Nordenfelt, S.; Harney E.; Stewardson, K.; et al.. Massive migration from the steppe was a source for Indo-European languages in Europe. *Nature* **2015,** *522*, 207-211.

Keller, A.; Graefen, A.; Ball, M,; Matzas, M.; Boisguerin, V.; Maixner, F.; Leidinger, P.; Backes, C.; Khairat R.; Forster, M.; et al. New insights into the Tyrolean Iceman's origin and phenotype as inferred by whole-genome sequencing. *Nat. Commun.* **2012,** *3*, 698.

Lazaridis, I.; Nadel, D.; Rollefson, G.; Merrett, D. C.; Rohland, N.; Mallick, S.; Fernandes, D.; Novak, M.; Gamarra, B.; Sirak, K.; et al. Genomic insights into the origin of farming in the ancient Near East. *Nature* **2016**, *536*, 419-424.

Lazaridis, I.; Patterson, N.; Mittnik, A.; Renaud, G.; Mallick, S.; Kirsanow, K.; Sudmant, P. H.; Schraiber, J. G.; Castellano, S.; Lipson, M.; et al. Ancient human genomes suggest three ancestral populations for present-day Europeans. *Nature* **2014***,* *513*, 409-413.

Olalde, I.; Allentoft. M.E.; Sanchez-Quinto, F.; Santpere, G.; Chiang, C. W.; DeGiorgio, M.; Prado-Martinez, J.; Rodríguez, J. A.; Rasmussen, S.; Quilez, J.; et al. Derived immune and ancestral pigmentation alleles in a 7,000-year-old Mesolithic European. *Nature* **2014,** *507*, 225-228.

Peng, Q.; Schork. N. J.; Wilhelmsen, K. C.; Ehlers, C. L. Whole genome sequence association and ancestry‐informed polygenic profile of EEG alpha in a Native American population. *Am. J. Med. Genet. B Neuropsychiatr. Genet.* **2017,** *174*, 435-450.

Raghavan, M.; DeGiorgio, M.; Albrechtsen, A.; Moltke, I.; Skoglund, P.; Korneliussen, T. S.; Grønnow, B.; Appelt, M.; Gulløv, H. C.; Friesen, T. M.; et al. The genetic prehistory of the New World Arctic. *Science* **2014** *345*, 1255832.

Schiffels, S.; Haak, W.; Paajanen, P.; Llamas, B.; Popescu, E.; Loe, L.; Clarke, R.; Lyons, A.; Mortimer, R.; Sayer, D.; et al. Iron Age and Anglo-Saxon genomes from East England reveal British migration history. *Nat. Commun*. **2016**, *7*, 10408.

Seguin-Orlando, A.; Korneliussen, T. S.; Sikora, M.; Malaspinas, A. S.; Manica, A.; Moltke, I.; Albrechtsen, A.; Ko, A.; Margaryan; A.; Moiseyev, V.; et al. Paleogenomics, Genomic structure in Europeans dating back at least 36,200 years. *Science* **2014***,* *346*, 1113-1118.

Skoglund, P.; Malmström, H.; Omrak, A.; Raghavan, M.; Valdiosera, C.; Günther, T.; Hall, P.; Tambets, K.; Parik, J.; Sjögren, K. G.; et al. Genomic diversity and admixture differs for Stone-Age Scandinavian foragers and farmers. *Science* **2014**, *344***,** 747-750.

© 2018 by the authors. Submitted for possible open access publication under the
terms and conditions of the Creative Commons Attribution (CC BY) license (http://creativecommons.org/licenses/by/4.0/).
